# Efficient Neutral Nitrate-to-Ammonia Electrosynthesis Using Synergistic Ru-Based Nanoalloys on Nitrogen-Doped Carbon

**DOI:** 10.1007/s40820-025-01896-w

**Published:** 2025-09-15

**Authors:** Lisi Huang, Pingzhi Zhang, Xin Ge, Bingyu Wang, Jili Yuan, Wei Li, Jian Zhang, Baohua Zhang, Ozge Hanay, Liang Wang

**Affiliations:** 1 Department of Polymer Materials and Engineering, College of Materials & Metallurgy, Guizhou University, Huaxi District, Guiyang, 550025 People’s Republic of China; 2 School of Chemistry and Materials Science, Hunan Agricultural University, Changsha, 410128 People’s Republic of China; 3 Institute of Nanochemistry and Nanobiology, School of Environmental and Chemical Engineering, Shanghai University, 99 Shangda Road, Baoshan District, Shanghai, 200444 People’s Republic of China; 4 Department of Chemical Engineering, School of Environmental and Chemical Engineering, Shanghai University, 99 Shangda Road, Shanghai, 200444 People’s Republic of China; 5 State Key Laboratory of Green Pesticide, Center for R&D of Fine Chemicals of Guizhou University, Guiyang, 550025 People’s Republic of China; 6 Department of Environmental Engineering, Faculty of Engineering, Firat University, 23119 Elazig, Turkey

**Keywords:** Synergic effect, Selective etching, Nanoalloys, Porous nitrogen-doped carbon, Neutral NH_3_ electrosynthesis

## Abstract

Electrocatalytic nitrate reduction reaction (NO_3_RR) represents a sustainable and environmentally benign route for ammonia (NH_3_) synthesis. However, NO_3_RR is still limited by the competition from hydrogen evolution reaction (HER) and the high energy barrier in the hydrogenation step of nitrogen-containing intermediates. Here, we report a selective etching strategy to construct RuM nanoalloys (M = Fe, Co, Ni, Cu) uniformly dispersed on porous nitrogen-doped carbon substrates for efficient neutral NH_3_ electrosynthesis. Density functional theory calculations confirm that the synergic effect between Ru and transition metal M modulates the electronic structure of the alloy, significantly lowering the energy barrier for the conversion of *NO_2_ to *HNO_2_. Experimentally, the optimized RuFe-NC catalyst achieves 100% Faraday efficiency with a high yield rate of 0.83 mg h^−1^ mg_cat_^−1^ at a low potential of − 0.1 V vs. RHE, outperforming most reported catalysts. In situ spectroscopic analyses further demonstrate that the RuM-NC effectively promotes the hydrogenation of nitrogen intermediates while inhibiting the formation of hydrogen radicals, thereby reducing HER competition. The RuFe-NC assembled Zn-NO_3_^−^ battery achieved a high open-circuit voltage and an outstanding power density and capacity, which drive selective NO_3_^−^ conversion to NH_3_. This work provides a powerful synergistic design strategy for efficient NH_3_ electrosynthesis and a general framework for the development of advanced multi-component catalysts for sustainable nitrogen conversion.

**Supplementary Information:**

The online version contains supplementary material available at 10.1007/s40820-025-01896-w.

## Highlights

- A selective etching strategy was developed to construct a serious of RuM nanoalloys (M = Fe, Co, Ni, Cu) uniformly dispersed on porous nitrogen-doped carbon.
- It has been demonstrated that RuM nanoalloys would present the enhancement synergic effect on significantly improve the kinetic of *NO_2_ conversion to *HNO_2_, which achieves efficient neutral NH_3_ electrosynthesis at more positive potential.

**Supplementary Information:**

The online version contains supplementary material available at 10.1007/s40820-025-01896-w.

## Graphical abstract

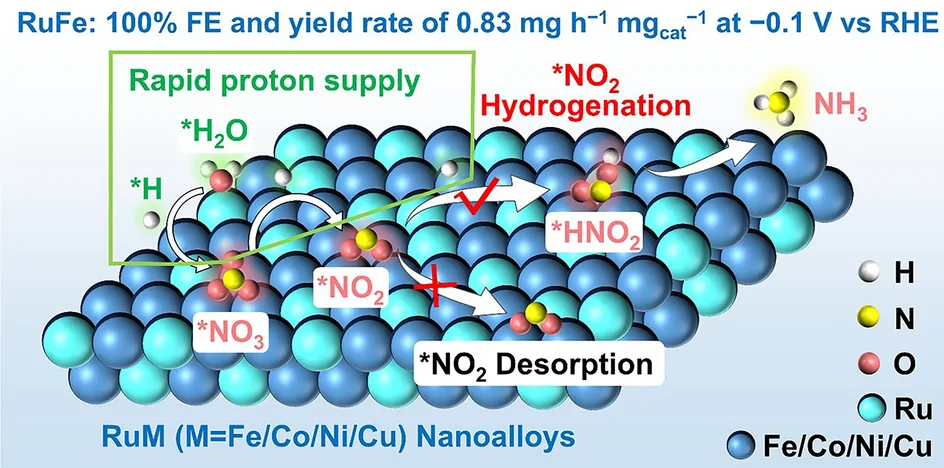

## Introduction

Ammonia (NH_3_) serves as a crucial chemical feedstock with indispensable applications in agricultural and industrial sectors, particularly in fertilizer production and emerging energy storage technologies [1–3]. Traditionally, NH_3_ synthesis relies on the Haber–Bosch process, which operates under extreme temperatures (over 500 °C) and high pressures (over 200 atm) [4–6], leading to substantial energy consumption and considerable carbon emissions [7–9]. As a sustainable alternative [10], the electrochemical nitrate reduction reaction (NO_3_RR) offers the potential for decentralized and low-temperature NH_3_ production due to its relatively weak N–O bonding energy of NO_3_^−^ [11, 12]. However, NO_3_RR yet faces challenges stemming from its complex proton coupling with electron transfer and multi-step hydrogenation of NO_3_^−^ [13, 14]. These processes generally involve the desorption of various nitrogen-containing intermediates caused by suddenly enhanced energy barrier, ultimately resulting in unsatisfactory NH_3_ selectivity and activity even under large applied potential because of the occurrence of hydrogen evolution reaction (HER) [15, 16]. Therefore, the development of efficient catalysts to reduce the reaction energy barrier and inhibit HER is critical to realize the high selectivity and activity for NH_3_ production.

To date, various electrocatalysts have been explored to address these limitations, such as single-atom catalysts [17–19], alloy [20–25], and transition metal compounds [26]. Most reported strategies primarily [16, 27–29] focus on facilitating the rate-determining step (RDS) of NO_3_^−^ → NO_2_^−^ to break in reaction kinetics bottleneck. However, the subsequent hydrogenation process of NO_2_^−^ → HNO_2_^−^, which involves further proton-coupled electron transfers, still requires in-depth investigation. Particularly under neutral conditions, the insufficient supply of active *H species restricts the hydrogenation of NO_2_^−^, leading to excessive accumulation of NO_2_^−^ intermediates and hindering their subsequent conversion to NH_3_ [30, 31]. For example, in the Ni_3_N nanosheet array intimately decorated with Cu nanoclusters (NF/Ni_3_N–Cu) catalyst, *H species generated at the Ni active sites of Ni_3_N are transferred to Cu sites via a reverse hydrogen spillover process mediated by interfacial Ni–N-Cu bridge bonds. This long-range hydrogen transfer pathway requires overcoming energy barriers across heterojunction interfaces, resulting in significantly sluggish kinetics [32]. Although the Cu_1_Co_5_ alloy alleviates the kinetic bottleneck through optimized hydrogen transfer pathways, its water dissociation efficiency remains insufficient for continuous *H generation [20]. Similarly, RhSb alloy nanoflowers leverage p-d orbital coupling to inhibit HER and enhance *H generation efficiency, but exhibit inadequate adsorption capacity for NO_3_^−^ [33]. In general, noble metals enhance active *H species supply by promoting water dissociation [34], whereas transition metals optimize adsorption of intermediates through electron distribution modulation. Therefore, constructing noble metal-transition metal alloy catalysts represents an effective strategy to overcome the dual challenges of sluggish hydrogenation and HER competition in NO_3_RR.

Herein, we report a selective etching strategy for constructing RuM (M = Fe, Co, Ni, Cu) nanoalloys anchored on nitrogen-doped carbon supports. The synergic effect of alloying overcomes the challenge of the sluggish NO_2_^−^ hydrogenation and the insufficient supply of *H species under neutral conditions. Density functional theory (DFT) calculations reveal that the incorporation of transition metals modulates electron distribution in Ru, thereby lowering the hydrogenation energy barrier and promoting the formation of essential *H species. Electrochemical tests further verify that RuM-NC enhances the NO_2_^−^ reduction and effectively suppresses HER. Furthermore, in situ spectroscopy further demonstrates that, compared with Ru-NC, RuM-NC exhibits distinct characteristic peaks of HNO_2_ adsorption, which provides direct experimental evidence for the reduction of the reaction energy barrier. Moreover, RuFe-NC in the assembled Zn-NO_3_^−^ battery achieved an outstanding performance of power density and NH_3_ production, and realized NO_3_^−^ recovery to NH_3_ powered by Zn-NO_3_^−^ battery. This work presents a strategy for developing efficient NO_3_RR catalysts and promotes a fundamental understanding of the mechanism by which the synergic effect of alloying lowers the energy barrier of reaction under neutral conditions.

## Experimental Section

### Synthesis of RuM (M = Fe, Co, Ni, Cu)-NC

Typically, 10 g of urea is added to a vial (with a capacity of 40 mL) and heated to 150 °C to melt it into a transparent liquid. Then, 400 mg of PEG 4000 is added under stirring. Subsequently, 20 g of NaCl, 0.1 g of RuCl_3_, and 0.532 g of FeCl_3_ are added and ground for 15 min. The melted urea is poured into an alumina crucible and subjected to pyrolysis in a tube furnace at 790 °C under an argon atmosphere, with a heating rate of 10 °C min^−1^ for 1.5 h, followed by natural cooling to room temperature. Before heating, the tube furnace is purged with nitrogen for 30 min to remove oxygen. After naturally cooling to room temperature, the product is washed three times with deionized water, with each wash involving stirring for 30 min. The product is then heated to 80 °C and treated with 2 M HCl for 24 h. Finally, the product is dried overnight under vacuum at 60 °C to obtain the final product. The preparation processes for RuCo-NC, RuNi-NC, RuCu-NC, and Ru-NC are similar to that of RuFe-NC, except for the differences in the masses of the metal salts used.

### Computational Details

All calculations were performed using the Vienna Ab initio Simulation Package (VASP) based on DFT [35]. The generalized gradient approximation (GGA) was employed to describe the exchange–correlation interaction, utilizing the Perdew–Burke–Ernzerhof (PBE) functional [36]. The core electrons were treated using the projector augmented wave (PAW) method, which effectively describes the influence of core electrons on the valence electron density [37]. A plane-wave kinetic energy cutoff of 400 eV was used for all calculations. During structure relaxation, the energy and force convergence criteria were set to less than 10^–4^ eV and 0.01 eV Å^−1^, respectively. To sample the Brillouin zone, a Monk horst–Pack mesh of 2 × 2 × 1 k-points was used for both geometry optimization and electronic structure calculations [38]. A vacuum layer of 18 Å was applied to ensure sufficient separation between periodic images. Van der Waals interactions were included using Grimme’s DFT-D3 dispersion correction scheme [39].The hydrogen electrode model was employed in conjunction with the computational hydrogen electrode (CHE) approach, which is widely used for evaluating the Gibbs free energy change (ΔG) of each elemental step in electrochemical reactions. The Gibbs free energy change for adsorption (Δ*G*_ads_) is given by the following equation [40]:

1 $$\Delta G_{{{\mathrm{ads}}}} = \Delta E_{{{\mathrm{ads}}}} + \Delta ZPE - T\Delta S + eU$$

where Δ*E*_ads_ represents the adsorption binding energy, Δ*ZPE* and Δ*S* are the changes in zero-point energy and entropy, respectively, *T* is the temperature, *U* is the applied potential at the electrode, and *e*^−^ is the electron charge.

## Results and Discussion

### Synergic Effect Between Ru and M in NO_3_RR

**Fig. 1:**
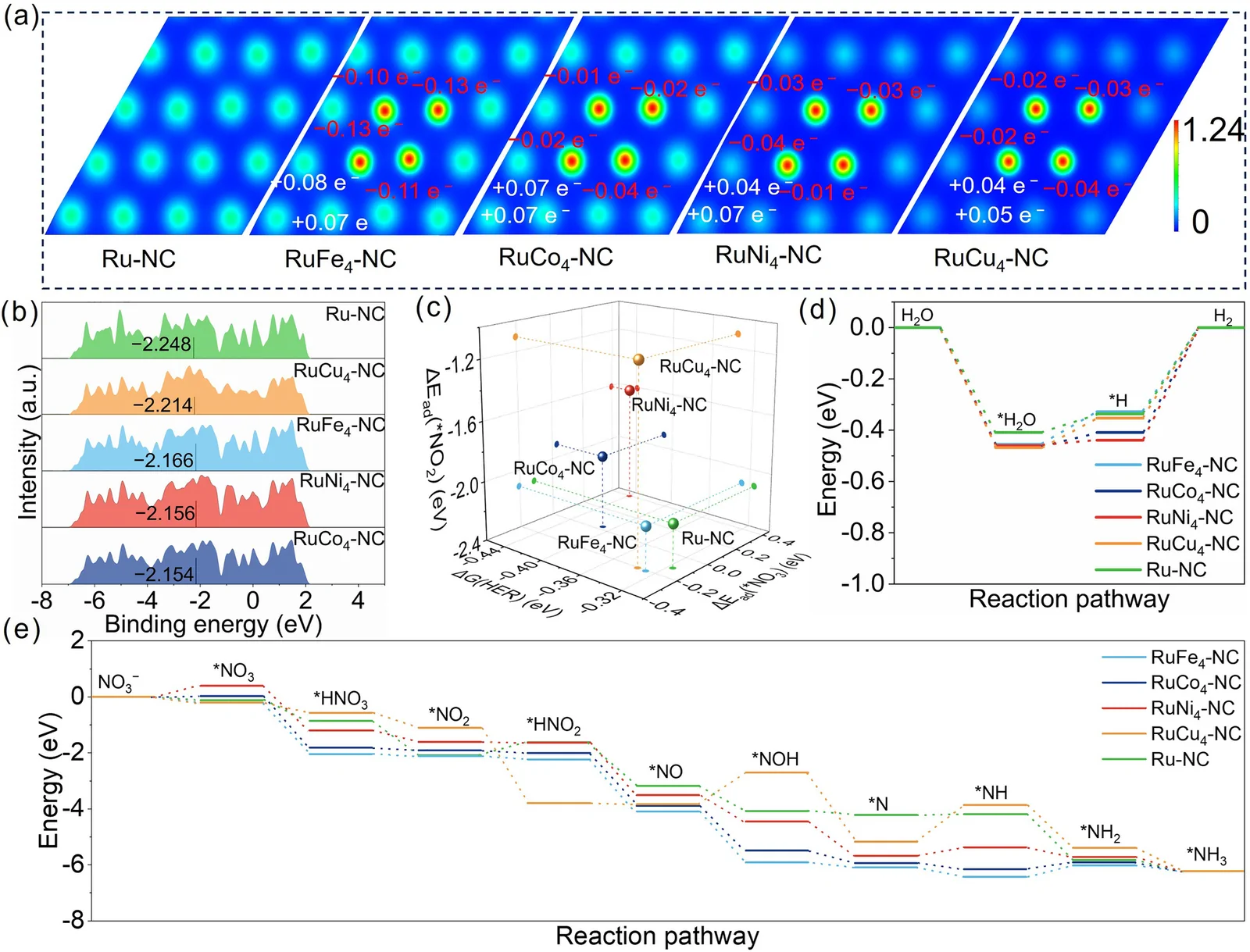
Theoretical simulation of electrochemical NO_3_RR. **a** 2D slice passing through the first layer atoms of Fe, Co, Ni, and Cu-doped Ru surfaces and pure Ru. The surface value in **a** is 0.1 e^−^/Bohr^3^. Red and blue in the charge density difference plot represent electron accumulation and depletion. **b** PDOS of the d-orbital of RuM_4_-NC and Ru-NC. **c** Adsorption energy of *NO_3_, *NO_2_, and Gibbs free energy for H_2_ formation on RuM_4_-NC and Ru-NC. **d** Reaction energy changes of HER on the Ru−M_4_ site and pure Ru site. **e** Reaction free energy diagrams of NO_3_^−^ to NH_3_

To elucidate the synergic effect between Ru and transition metals (M) in NO_3_RR, DFT calculations were carried out on RuM nanoalloys supported by defective nitrogen-doped carbon. The structural configurations of the Ru–M and Ru sites were constructed on M-doped Ru (101) and Ru (101) surfaces, respectively (Fig. S1). Charge density analysis reveals electron depletion at M sites near Ru, indicating that RuM nanoalloys effectively modulate electron transfer between Ru and M sites (Fig. 1a). Subsequently, projected density of states (PDOS) analysis was further performed to investigate the influence of the synergic effect between Ru and M on d-orbital energy levels (Fig. 1b). The calculated d-band centers of RuFe_4_-NC, RuCo_4_-NC, RuNi_4_-NC, RuCu_4_-NC, and Ru-NC are − 2.166, − 2.154, − 2.156, − 2.214, and − 2.248 eV, respectively, revealing that the synergic effect between Ru and M induce a positive shift in the d-band center toward the Fermi level, which facilitates electron migration during the catalytic process. The adsorption energies of *NO_3_ (Δ*E*_ad_ (*NO_3_)), *NO_2_ (Δ*E*_ad_ (*NO_2_)), and *H (Δ*G* (HER)) were employed as descriptors to evaluate the performance of electrocatalytic NO_3_RR (Fig. 1c). It is well known that the strong binding of *NO_3_ and *NO_2_ carries the risk of catalyst poisoning, while the weak binding hinders the activation and hydrogenation of intermediates [41, 42]. Meanwhile, the moderate adsorption of *H inhibits the competitive HER [43]. From the adsorption energy schematics of RuM nanoalloys, the synergic effect between Ru and M (M = Fe, Co, Cu) produces moderate adsorption energies of *NO_3_ and *NO_2_ while inhibiting HER, which may be promising candidate catalyst. Therefore, these results demonstrate that synergic effects between Ru and M modulate the electron transfer, thereby facilitating intermediate activation and suppressing HER, while also potentially enhancing NH_3_ synthesis performance.

The free energy associated with the formation of *H species on Ru–M sites and Ru sites is revealed in Figs. 1d and S2. The adsorption of H_2_O to form *H_2_O is thermodynamically spontaneous. Notably, Ru–M sites exhibit more negative for energy barrier the formation of *H_2_O from H_2_O compared to Ru sites (− 0.409 eV), demonstrating higher thermodynamic spontaneity. Moreover, the formation of H_2_ from *H on Ru–M sites requires a higher energy than the Ru sites (0.316 eV), demonstrating that the synergic effect between Ru and M facilitate the formation of *H_2_O while suppressing the *H → H_2_ pathway. These results demonstrate a higher coverage of *H on RuM-NC compared to Ru-NC, favoring efficient hydrogenation of nitrogen-containing intermediates in NO_3_RR and inhibiting HER (Fig. 1d). The energy barrier for key steps further validates the synergic effect between Ru and M (Figs. 1e and S3–S7). For the rate-limiting step from *NO_2_ to *HNO_2_, the energy barrier at the Ru–M site was lower than that at the Ru site (0.464 eV), suggesting that the synergic effect between Ru and M reduced the energy barrier for the production of NH_3_. Among them, RuCu_4_-NC exhibits the highest spontaneity for the *NO_2_ → *HNO_2_ conversion, but it exhibits two larger energy barrier (*NO → *NOH and *N → *NH), which leads to a decrease in the activity of RuCu_4_-NC. Moreover, *NO → *NH_3_ conversion is a more spontaneous at Ru–M sites in contrast to Ru sites (Fig. 1e). In addition, doping Ru with an M atom still reduces the energy barrier for the *NO_2_ → *HNO_2_ conversion, and the extent of this reduction becomes more pronounced as the number of doped atoms increases (Figs. S8–S17, Supplement Text S1, S2).Taken together, these results elucidate that synergistic interaction between Ru and transition metals reduce the energy barrier of *NO_2_ → *HNO_2_ and promote thermodynamically favorable conversions of nitrogen-containing intermediates, which further demonstrates that the combination of Ru and transition metals in Ru-based nanoalloys is a promising candidate for efficient.

### Material Synthesis and Characterizations

**Fig. 2:**
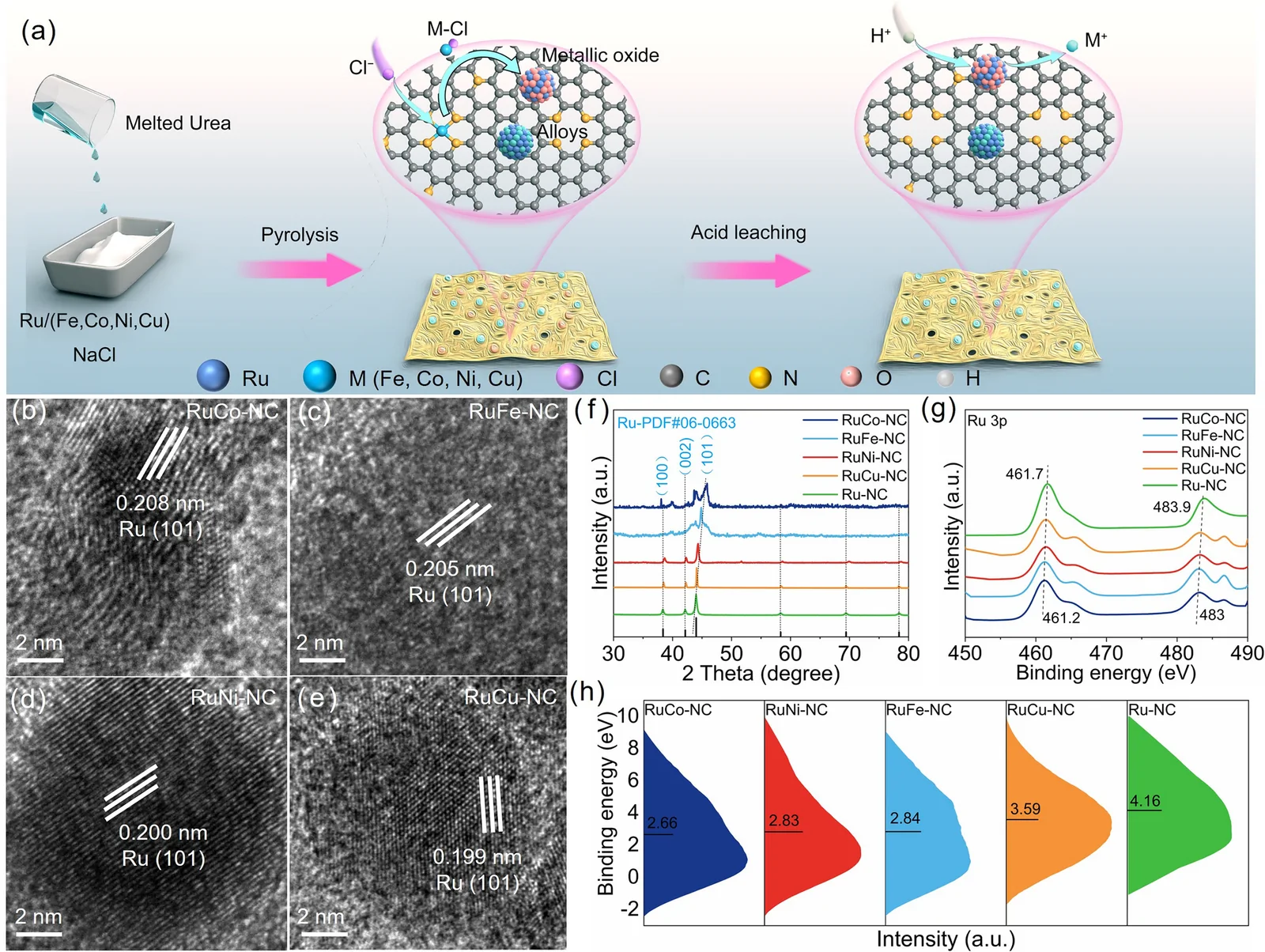
Preparation strategy and structural characterization of catalyst. **a** Schematic diagram of catalyst preparation. High-resolution TEM images of **b** RuCo-NC, **c** RuFe-NC, **d** RuNi-NC and **e** RuCu-NC. **f** X-ray diffraction pattern. **g** High-resolution XPS Ru 3*p* spectra. **h** d-band center shifts are calculated by integrating XPS valence band spectra

A selective etching strategy, which primarily involves chloride-ion-assisted induction and in situ formation of metal oxides followed by pyrolytic reduction (Figs. S18–S20), has been successfully employed to fabricate RuM-NC nanoalloys uniformly anchored on nitrogen-doped carbon substrates (Fig. 2a, Supplement Text S3). This strategy enables the controlled incorporation of transition metals (M = Fe, Co, Ni, Cu) into the Ru lattice during thermal treatment.

The high-resolution transmission electron microscopy (HR-TEM) analysis confirms the formation of RuM-NC nanoalloys with well-defined crystalline structures. The measured lattice spacing of 0.208, 0.205, 0.200, and 0.199 nm for RuCo-NC, RuFe-NC, RuNi-NC, and RuCu-NC nanoalloys, respectively, which can be assigned to Ru (101) plane (Fig. 2b–e), indicating successful alloying and subtle modulation of lattice parameters [44]. The progressive decrease in lattice spacing with increasing incorporation of smaller-radius transition metals suggests a lattice contraction effect, further verifying the formation of Ru-based bimetallic alloys. These nanoalloys are well-dispersed distribution on carbon substrates without obvious agglomeration. Meantime, the atomic-resolution energy-dispersive X-ray spectroscopy (EDS) element mapping image of RuM-NC demonstrates homogeneous distribution of Ru and transition metal within the alloy nanoparticles, indicating successful doping within the Ru (101) plane (Fig. S21, Supplement Text S4) [45, 46]. The occurrence of the Ru diffraction peaks in the XRD of the pyrolysis sample (Fig. S18) confirms that ruthenium is completely reduced in pyrolysis and that the transition metal existed in an oxidized state. After acid etching, the transition metal oxides are removed and the final product is a Ru-based alloy. Moreover, a slight right-shift and broadening of the (101) diffraction peak in RuM-NC compared to pristine Ru-NC are observed (Fig. 2f). This shift reflects lattice contraction due to the incorporation of transition metals with smaller atomic radius, while the peak broadening indicates reduced crystallite size and increased alloying degree. Notably, the minor extra peaks observed in the analysis can be attributed to glitch signals upon careful comparison (Fig. S22), which do not interfere with the primary structural assignments. Therefore, these results confirm the successful synthesis of RuM-NC nanoalloys with tunable lattice structures and uniform metal distribution on the conductive carbon substrates, laying a solid foundation for subsequent electrocatalytic investigations.

The porous structure of the RuM-NC is confirmed by scanning electron microscopy (SEM) images, revealing an open-pore network being beneficial for mass transport during electrocatalysis (Fig. S23). Moreover, the X-ray photoelectron spectroscopy (XPS) spectra further confirm the successful incorporation of transition metals (M = Co, Fe, Ni, Cu) into the Ru lattice. Fig. S24 and Tables S1–S4 show that M diffraction peak can be observed, indicating the formation of bimetallic RuM structures. The high-resolution XPS spectra (Figs. S25–S29) and the EDS elemental mapping of RuM-NC (Fig. S21) confirm the successful anchoring of RuM on porous nitrogen-doped carbon substrates (Supplement Text S4). Additionally, Raman spectroscopy reveals the enhanced G-band intensity of RuM-NC relative to Ru-NC (Fig. S30) [47, 48], suggesting an increased graphitization degree in the carbon substrates, which may contribute to improved electronic conductivity.

The high-resolution XPS Ru 3*p* spectra exhibit two main peaks at positions 461.2 and 483 eV (Fig. 2g). Notably, upon alloying with transition metals, the Ru 3*p* binding energies in RuM-NC exhibit a consistent downshift compared to Ru-NC, demonstrating the electron transfer from M to Ru. This trend aligns with the previously observed lattice contraction in the XRD patterns and is further corroborated by the shift in the surface valence band d-band center, as illustrated in Fig. 2h. The centers of gravity for RuM-NC gradually shifted from M to Ru, suggesting a charge redistribution across the alloy interface, with Ru sites becoming electron-enriched and M sites acquiring localized positive charge density [49]. Such electron rearrangement is expected to promote preferential NO_3_^–^ adsorption on M sites and facilitate optimized water activation at electron-rich Ru centers. Therefore, these results suggest that through a selective etching strategy, the doping of different transition metals effectively regulated both the electronic structure and local coordination environment, which are key to enhancing the electrocatalytic performance in NO_3_RR.

### Electrocatalytic NO_3_^−^ Reduction Performance

**Fig. 3:**
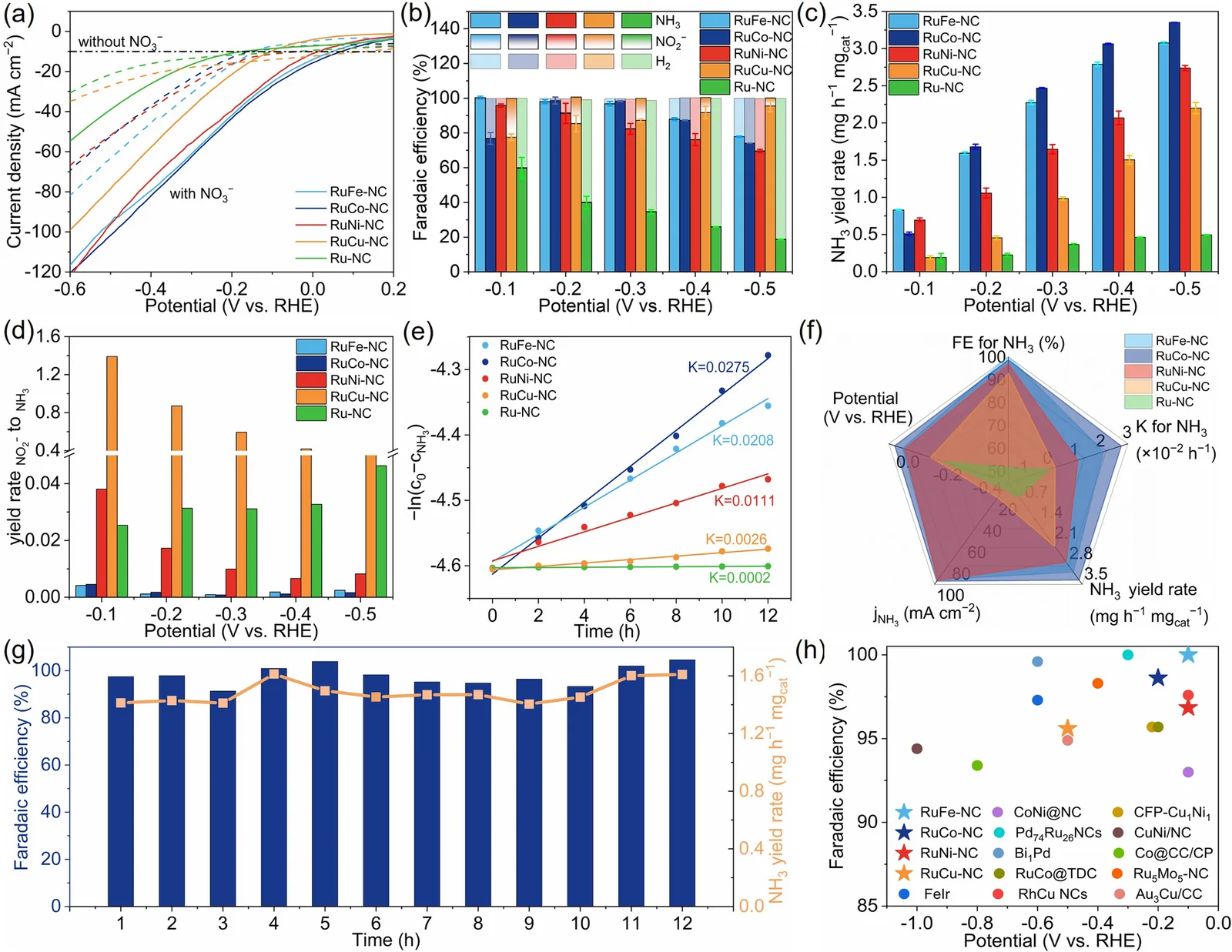
Evaluation of electrocatalytic NO_3_^−^to NH_3_ performance. **a** LSV curves of RuFe-NC, RuCo-NC, RuNi-NC, RuCu-NC, and Ru-NC nanoalloys in 0.5 M K_2_SO_4_ with and without 0.1 M NO_3_^−^. **b** FE, **c** NH_3_ yield rates, and **d** production ratios of NO_2_^−^ to NH_3_ of RuFe-NC, RuCo-NC, RuNi-NC, RuCu-NC, and Ru-NC at various potentials. **e** Kinetic constants (k) for NO_3_^−^ reduction to NH_3_. **f** Radar plot for the comparison of FE for NH_3_, kinetic constant for NO_3_^−^ reduction (k), NH_3_ yield rate, partial current density (jNH_3_), and the overpotential of NO_3_^−^ electroreduction at the current density of 10 mA cm^−2^. **g** Consecutive recycling test at − 0.2 V vs. RHE over RuCo-NC. **h** Comparison of the NH_3_ FE over RuFe-NC, RuCo-NC, RuNi-NC, and RuCo-NC with the reported alloy electrocatalysts

Electrocatalytic NO_3_RR performance was evaluated using a three-electrode H-type cell under ambient conditions. The reaction products were comprehensively analyzed through a detection approach: gaseous H_2_ was monitored in real-time using online gas chromatography, while liquid products were quantitatively determined through post-reaction analysis (Figs. S31 and S32). To investigate NO_3_RR activity, linear sweep voltammetry (LSV) was carried out in 0.5 M K_2_SO_4_ with and without 0.1 M NO_3_^−^ (Fig. 3a). The results demonstrate that the introduction of NO_3_^−^ into the 0.5 M K_2_SO_4_ electrolyte significantly enhances the current density for RuM-NC, confirming the effective occurrence of the NO_3_RR process. The onset potentials follow the trend: RuCo-NC (0.07 V vs. RHE) > RuFe-NC (0.03 V vs. RHE) > RuNi-NC (0.02 V vs. RHE) > RuCu-NC (− 0.11 V vs. RHE) > Ru-NC (− 0.78 V vs. RHE), suggesting that alloying with transition metals considerably promotes the reaction kinetics. This trend essentially corresponds to the more positive shift of the d-band center (Fig. 2h), underscoring the critical role of electronic modulation via M doping. Particularly, RuCo-NC, RuFe-NC, RuNi-NC, and RuCu-NC exhibit significantly higher onset potentials compared to Ru-NC, indicating that the synergic effect of between transition metals and Ru in RuM-NC exerts a positive influence on the NO_3_RR. Moreover, the current densities of the obtained RuM-NC and Ru-NC were normalized by the electrochemical surface area (ECSA) determined based on electric double-layer capacitance (C_dl_) and specific capacitance (C_s_) measurements (Figs. S33–S35) [50–52]. The ECSA normalized current density of RuM-NC was higher than that of Ru-NC, which confirmed that the synergistic effect of alloying could enhance the intrinsic activity of NH_3_ synthesis. To verify the nitrogen origin in synthesized NH_3_ [53, 54], we performed isotope labeling experiments using ^14^N and ^15^N during NO_3_RR (Fig. S36). Quantification via ^1^H NMR and UV–Vis absorption revealed comparable NH_3_ yields for both isotopes, confirming that NH_3_ derives from electro reduced NO_3_^−^.

To investigate the influence of transition metals in RuM-NC on the NO_3_RR performance, ammonia selectivity was further evaluated across a range of applied potentials (− 0.1 to − 0.5 V vs. RHE). As depicted in Fig. 3b, the NH_3_ Faradaic efficiency (FE) at 100% for RuFe-NC at − 0.1 V vs. RHE, followed by RuCo-NC (98.62%, − 0.2 V vs. RHE), RuNi-NC (96.85%, − 0.1 V vs. RHE), RuCu-NC (95.60%, − 0.5 V vs. RHE), Ru-NC (51.42%, − 0.1 V vs. RHE), NC (9.84%, − 0.4 V vs. RHE) and RuFe-NC at 0 V, 0.1 V vs. RHE electrochemical testing revealed a decrease in NH_3_ FE, indicating that it was the optimal potential for RuFe-NC at − 0.1 V (Figs. S37 and S38). Notably, the H_2_ selectivity over Ru-NC increases with more negative potentials, whereas RuM-NC exhibits significantly high NH_3_ selectivity and with suppressed H_2_ generation (Fig. 3b). These observations highlight the inhibitory effect of the competing HER in Ru-NC, which is effectively mitigated through transition metal alloying. The experimentally determined selectivity trend is highly in alignment with our theoretical predictions (Fig. 1e), supporting the proposed synergistic interaction between Ru and M in tuning the adsorption energetics of NO_3_^−^ intermediates and suppressing HER.

The NH_3_ yield rate exhibited a gradual increase as the potential became more negative (Figs. 3c and S37). At − 0.5 V vs. RHE, the NH_3_ yield followed the order: RuCo-NC (highest, 3.35 mg h^−1^ mg_cat_^−1^) > RuFe-NC (3.08 mg h^−1^ mg_cat_^−1^) > RuNi-NC (2.74 mg h^−1^ mg_cat_^−1^) > RuCu-NC (2.20 mg h^−1^ mg_cat_^−1^) > Ru-NC (0.49 mg h^−1^ mg_cat_^−1^) > NC (0.00482 mg h^−1^ mg_cat_^−1^). Compared with Ru-NC, the production ratio of NO_2_^−^ to NH_3_ for RuFe-NC, RuCo-NC, and RuNi-NC decreased, while it increased for RuCu-NC (Fig. 3d). Notably, RuFe-NC and RuCo-NC exhibited the lowest production ratio of NO_2_^−^ to NH_3_, further highlighting their superior selectivity toward complete NO_3_^−^ reduction to NH_3_. Furthermore, in neutral electrolytes in the range of 0.05–0.5 M NO_3_^−^, RuFe-NC and RuCo-NC still achieves a NH_3_ FE exceeding 80% at − 0.4 V vs. RHE in 0.05 M NO_3_⁻ (Figs. S39 and S40), demonstrating excellent catalytic performance under practical conditions.

During the electrocatalytic NO_3_RR process, the kinetic constants (K) for RuM-NC and Ru-NC exhibit a pseudo-zero-order dependence on NO_3_^−^ concentration over a 12-h (Fig. 3e), suggesting surface-limited reaction behavior. Among these, RuM-NC demonstrates a higher reaction rate constant than Ru-NC (0.0002 h^−1^), underscoring the beneficial effect of transition metal doping on the reaction kinetics. As shown in Fig. S41, RuCo-NC exhibits superior conductivity compared to the other RuM-NC, indicating its ability to accelerate electron transfer during NO_3_RR, thereby contributing to its superior catalytic performance. Radar plot further demonstrates that RuCo-NC achieves the highest NH_3_ electrosynthesis performance among all tested catalysts, outperforming RuFe-NC, RuNi-NC, RuCu-NC, and Ru-NC (Fig. 3f). Consecutive recycling tests for NO_3_RR were conducted on RuCo-NC (Fig. 3g) and RuFe-NC (Fig. S42), suggesting nearly stable NH_3_ FE and yield rates, demonstrating excellent electrochemical durability. Post-electrolysis characterization confirmed the stability of the morphology and porous structure of RuM-NC. Notably, RuM-NC remained uniformly dispersed on the nitrogen-doped carbon substrates and retained good crystallinity (Fig. S43). Characterization after stability testing was also carried out, and the structure remained almost unchanged (Figs. S44 and S45). In addition, RuM-NC exhibited high HER selectivity under acidic (pH = 1) and alkaline (pH = 13) conditions, but still maintained high NH_3_ selectivity (Fig. S46). Compared to previously reported Ru-based nanoalloy electrocatalysts for NO_3_RR (Fig. 3h), RuFe-NC achieves the highest NH_3_ FE at a low potential of − 0.1 V vs. RHE, highlighting its exceptional neutral NO_3_RR activity. These findings demonstrate that the selective etching strategy employed in RuM-NC not only enhances NO_3_RR selectivity but also effectively suppresses the competing HER, thereby demonstrating the significant potential of synergistic interactions in advancing NO_3_RR.

### In Situ Observations and Mechanism Investigation

**Fig. 4:**
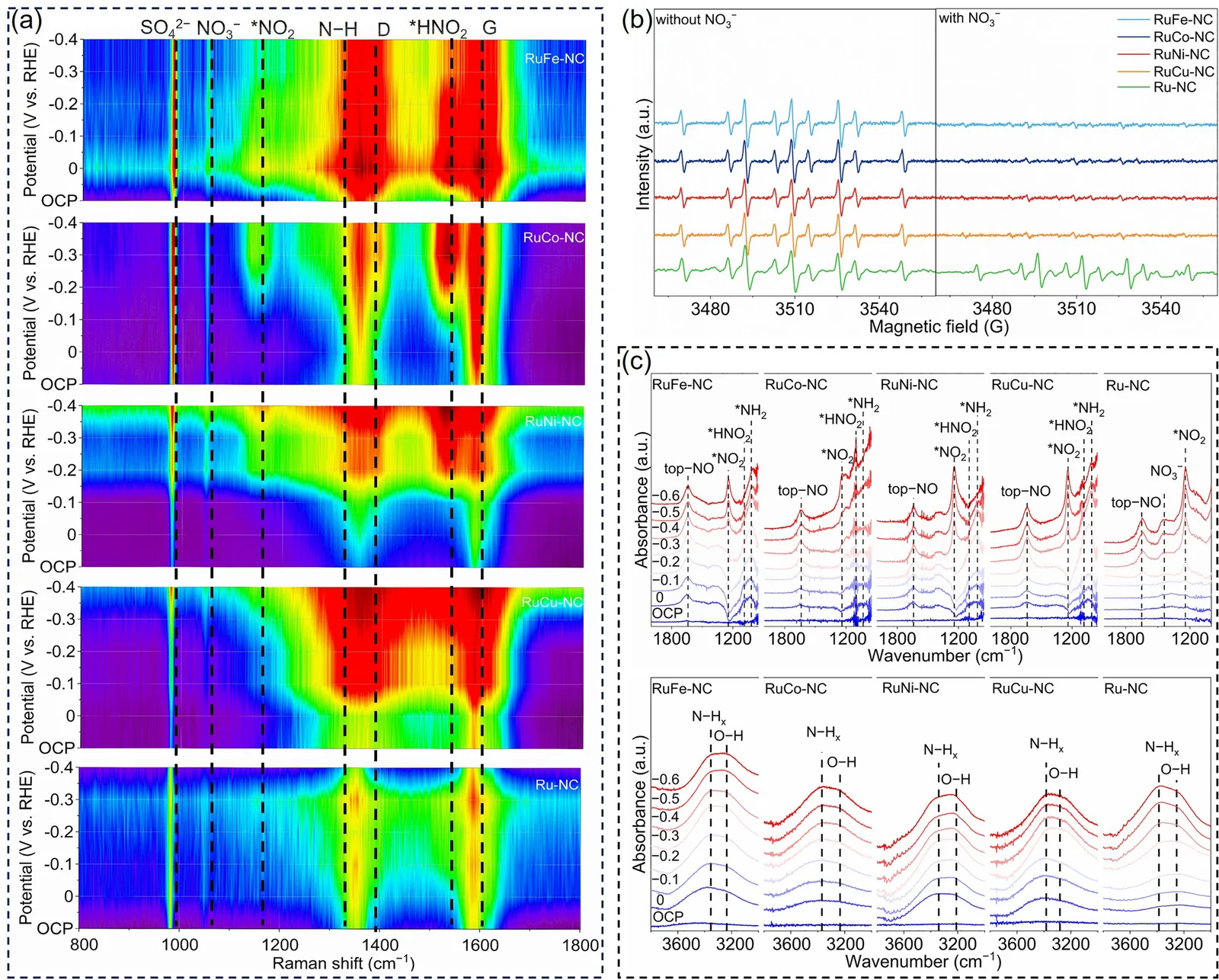
In situ observations. **a** In situ Raman spectra RuFe-NC, RuCo-NC, RuNi-NC, RuCu-NC, and Ru-NC. **b** In situ EPR spectra for ·H in 0.1 M NO_3_^−^ over RuFe-NC, RuCo-NC, RuNi-NC, RuCu-NC, and Ru-NC. **c** In situ ATR-SEIRAS spectra of RuFe-NC, RuCo-NC, RuNi-NC, RuCu-NC, and Ru-NC

To elucidate the mechanism of NO_3_RR, reaction intermediates with different potentials were captured by in situ Raman [55]. The Raman signals at 1381 and 1591 cm^−1^ correlated with the D and G bands of the carbon substrate [56, 57] (Figs. 4a and S30), while the peak at 1140 cm^−1^ is identified as adsorbed *NH_3_ [58]. Notably, the *NH_3_ peak of RuM(M = Fe, Co, Ni) is stronger than that of RuCu-NC, suggesting that less NH_3_ is adsorbed on RuCu-NC compared to other samples, which is consistent with its lower optimal selectivity for NH_3_ production (Fig. 3b). Furthermore, RuM-NC exhibits additional Raman characteristic peaks at approximately 1358 and 1528 cm^−1^ corresponding to *HNH and *HNO_2_ intermediates [59], which are absent in Ru-NC. These results indicate that the incorporation of transition metals enhances the adsorption and hydrogenation of nitrogenous intermediates via synergistic interaction with Ru, thereby promoting NO_3_RR kinetics and selectivity.

Complementary insights into hydrogen-involving steps were obtained utilizing electron spin resonance (ESR) spectroscopy with DMPO as the spin-trapping agent (Fig. 4b). In 0.5 M K_2_SO_4_ without NO_3_^−^, the DMPO-H signal of Ru-NC was the most pronounced compared with RuM-NC, indicating its strong hydrogen adsorption capability. Although RuM-NC catalysts still retain strong hydrogen adsorption capabilities, their DMPO-H signal intensities are markedly lower in the presence of NO_3_^−^, indicating a favorable shift toward selective *H utilization for NO_3_RR rather than HER (Fig. 4b). Notably, these observations correlate well with the electrochemical results in Fig. 3b, where Ru-NC exhibits dominant HER activity with increasing overpotential, while RuM-NC maintains high NH_3_ selectivity. Moreover, RuM-NC exhibit markedly lower ·H signals in a neutral electrolyte containing NO_3_^−^ (Fig. 4b), indicating that hydrogen atoms are effectively consumed in the hydrogenation of NO_3_RR intermediates, rather than recombining to form H_2_. These results indicate that in RuM-NC, *H is preferentially utilized for the hydrogenation of nitrogen intermediates during NO_3_RR, whereas in Ru-NC, *H promotes HER, thereby, the synergic effect between Ru and transition metals thus enable efficient NH_3_ production through a well-regulated reaction pathway.

To further elucidate the surface reaction processes and intermediates involved in NO_3_RR, in situ attenuated total reflective surface-enhanced infrared absorption spectroscopy (ATR-SEIRAS) was utilized to investigate the molecular-level behaviors of RuM-NC and Ru-NC. As shown at the top of Fig. 4c, for RuM-NC (M = Fe, Co, Ni), the *NO_2_ peak intensifies with increasingly negative potentials, indicating that the hydrogenation of *NO_2_ to *HNO_2_ is likely the rate-limiting step. Notably, the N–O antisymmetric tensile vibration of adsorbed *NO_2_ species at 1240 cm^−1^ are detected in RuM-NC [59, 60]. Although RuNi-NC also exhibits a stronger NO_2_ signal at specific potentials (e.g., − 0.4 V vs. RHE), RuCu-NC exhibits a more significant NO_2_ accumulation trend over a wider potential range (0 to − 0.6 V vs. RHE) (Fig. 4c). In addition, the NO_2_ signal intensity of RuCu-NC continues to increase at negative potential, while the signal of RuNi-NC tends to be saturated at lower potential. This dynamic change indicates that the RuCu-NC surface is more conducive to the stable adsorption of NO_2_, which coincides with its higher NO_2_^−^ selectivity (Fig. 3b). In contrast, the NO_2_ signal of RuFe-NC, RuCo-NC, and Ru-NC was weak, indicating that these catalysts have a low adsorption capacity for *NO_2_. Moreover, the presence of *HNO_2_ peaks in RuM-NC compare to Ru-NC suggests that the synergic effect between Ru and M prevents competing HER and promotes further hydrogenation of *NO_2_. In contrast, RuM-NC exhibits additional infrared bands absent in Ru-NC, particularly at approximately 1080 and 1028 cm^−1^ in the infrared spectrum, which are respectively attributed to the adsorption of the –N–O tensile vibration of *HNO_2_ and the –N–H bending vibration of *NH_2_ species [59, 61, 62]. The emergence of these bands indicates that RuM-NC facilitate the conversion of *NO_2_ to *HNO_2_ through synergic effects, suppressing undesired HER pathways and enabling preferential hydrogenation of nitrogen intermediates during NO_3_RR. Notably, the appearance of *HNO_2_ peaks in RuM-NC but not in Ru-NC highlights the crucial role of Ru–M synergy in modulating intermediate adsorption and hydrogenation.

Moreover, the onset potentials for the evolution of these vibrational features in RuM-NC are more positive compared to Ru-NC, indicating that RuM-NC exhibits better kinetics for NO_3_RR (Figs. 4c and 3e). This suggests that the introduction of transition metals into the Ru matrix not only modifies the electronic structure of active sites but also lowers the activation energy barrier for intermediate adsorption and hydrogenation steps. In addition to nitrogen-containing intermediates, RuM-NC also exhibits strong vibrational peaks corresponding to N–Hₓ at 3371 cm^−1^ and–O–H at 3236 cm^−1^ [63–65] (Fig. 4c). These results imply that RuM-NC promotes the dissociation of water, resulting in the generation of active *H species for NO_3_RR. Collectively, these findings demonstrate that the excellent synergistic interaction between Ru and the doped transition metal centers enhances NO_3_^−^ adsorption, promotes the dissociation of water to produce active hydrogen species, and directs the continuous hydrogenation step toward NH_3_ while suppressing HER.

To further highlight the practical potential of our catalysts, we have constructed a rechargeable Zn–NO_3_^−^ battery using a RuFe-NC cathode and a Zn sheet anode. This integrated system couples NO_3_RR with Zn oxidation reaction to simultaneously generate NH_3_ and electrical energy. As shown in Fig. S47a, the assembled battery delivered a high open-circuit voltage (OCV) of 1.483 V versus Zn^2+^/Zn. At current densities of 2, 5, and 7 mA cm^−2^, the calculated specific capacities of the RuFe-NC-based Zn-NO_3_^−^ battery were 674.4, 610, and 594.5 mAh g^−1^, respectively, demonstrating its superior electrocatalytic performance and energy conversion efficiency for NO_3_RR battery applications (Fig. S47b). It also demonstrates a power density of 10.16 mW cm^−2^ at 16 mA cm^−2^ and excellent cycling stability (Fig. S47c). Furthermore, discharge tests were conducted at various current densities (Fig. S47d), revealing that the output potential decreased with increasing current density but could be maintained when the current density was adjusted back, indicating excellent long-term electrochemical stability during energy output. The battery achieved a maximum NH_3_ FE of 92% with an NH_3_ production rate of 0.688 mg h^−1^ mg_cat_^−1^, outperforming most reported Zn–NO_3_^−^ batteries (Fig. S47e). Additionally, a timer was connected to our Zn–NO_3_^−^ battery to intuitively gauge its practical effect. As presented in Fig. S47f, it can work smoothly for 24 h without sign of depletion. These studies unveil the practical applicability of Zn-NO_3_^−^-based batteries with great energy and environmental significance.

## Conclusion

In summary, we proposed a selective etching strategy to engineer RuM nanoalloys uniformly anchored on porous nitrogen-doped carbon substrates. DFT calculations first confirm that the alloying of Ru with transition metals induces a positive shift of the d-band center, promoting electron transfer and thereby lowering the reaction energy barrier for key NO_3_RR steps. Guided by this theoretical insight, the synthesized RuM-NC exhibits exceptional NO_3_RR performance under neutral conditions. Systematic comparisons show a clear activity trend that decreases with the increase in the atomic number of transition metals: RuFe-NC > RuCo-NC > RuNi-NC > RuCu-NC > Ru-NC. Among them, while RuFe-NC achieves FE of 100% for neutral NH_3_ electrosynthesis, surpassing most of the previously reported electrocatalysts. In situ spectroscopic investigations further demonstrate that the synergic effect between Ru and M promotes the adsorption and hydrogenation of critical nitrogen-containing intermediates while concurrently suppressing the competitive HER. Moreover, RuFe-NC in the assembled Zn-NO_3_^−^ battery presented a high open-circuit voltage of 1.483 V, an impressive power density of 10.16 mW cm^−2^, and an outstanding capacity of 594.5 mAh g^−1^ at 7 mA mg_cat_^−1^, which can be applied in the environmental NO_3_^−^ conversion to NH_3_ powered by itself assembled Zn-NO_3_^−^ battery. This work highlights a generalizable strategy for tuning the electronic structure and reaction pathways of noble metal via transition metal alloying. The demonstrated synergistic modulation of intermediate adsorption and activation offers valuable design principles for next-generation multi-component electrocatalysts toward sustainable nitrogen conversion.

## Supplementary Information

Below is the link to the electronic supplementary material.

Supplementary file1 (DOCX 8996 KB)
