# Supplementary material for: Efficient Neutral Nitrate-to-Ammonia Electrosynthesis Using Synergistic Ru-Based Nanoalloys on Nitrogen-Doped Carbon
Source: Nanomicro Lett. 2025 Sep 15;18:66. doi: 10.1007/s40820-025-01896-w (PMC12436672; doi:10.1007/s40820-025-01896-w)
Supplement: Supplementary file 1 — Supplementary file1 (DOCX 8996 KB) [file 40820_2025_1896_MOESM1_ESM.docx]

Supporting Information for

**Efficient Neutral Nitrate-to-Ammonia Electrosynthesis Using Synergistic Ru-Based Nanoalloys on Nitrogen-Doped Carbon**

Lisi Huang^1, 5#^, Pingzhi Zhang^2#^, Xin Ge^1^, Bingyu Wang^1^, Jili Yuan^1^*, Wei Li^2^*, Jian Zhang^5^*, Baohua Zhang^4^, Ozge Hanay^6^, Liang Wang^3^*

^1^Department of Polymer Materials and Engineering, College of Materials & Metallurgy, Guizhou University, Huaxi District, Guiyang 550025, P. R. China

^2^School of Chemistry and Materials Science, Hunan Agricultural University, Changsha 410128, P. R. China

^3^Institute of Nanochemistry and Nanobiology, School of Environmental and Chemical Engineering, Shanghai University, 99 Shangda Road, Baoshan District, Shanghai 200444 P. R. China

^4^Department of Chemical Engineering, School of Environmental and Chemical Engineering, Shanghai University, 99 Shangda Road, Shanghai 200444, P. R. China

^5^State Key Laboratory of Green Pesticide, Center for R&D of Fine Chemicals of Guizhou University, Guiyang 550025, P. R. China

^6^Department of Environmental Engineering, Faculty of Engineering, Firat University, Elazig, 23119, Turkey

^#^Lisi Huang and Pingzhi Zhangcontributed equally to this work.

*Corresponding authors. E-mail: [jlyuan@gzu.edu.cn](mailto:jlyuan@gzu.edu.cn) (Jili Yuan); [weili@hunau.edu.cn](mailto:weili@hunau.edu.cn) (Wei Li); [jianzhang@gzu.edu.cn](mailto:jianzhang@gzu.edu.cn) (Jian Zhang); [wangl@shu.edu.cn](mailto:wangl@shu.edu.cn) (Liang Wang)

**S1 Chemicals**

Nafion solution (~ 5 wt.%) was purchased from Sigma-Aldrich. Iron (III) chloride hexahydrate (AR), Cobalt chloride hexahydrate (AR), Nickel chloride hexahydrate (AR), Copper (II) chloride dihydrate (AR), salicylic acid (AR, 99.5%), potassium hydroxide (KOH, AR, 96%), sodium citrate dihydrate (98%), potassium nitrate (KNO_3_, 99%), sodium nitrite (NaNO_2_, 99.99% metals basis), ammonium chloride (NH_4_Cl, ACS, 99.5%), potassium sulfate anhydrous (K_2_SO_4_, 99%), N (1-naphthyl) ethylenediamine dihydrochloride (AR, 98%), p-aminobenzene sulfonamide (AR, ≥ 99%), phosphoric acid (H_3_PO_4_, ACS, ≥ 85 wt.% in H_2_O, ρ = 1.70 g/mL), potassium nitrate-^15^N (99 atom%, ≥ 98.5%), ammonium chloride-^15^N (98 atom%, ≥ 98%), sodium hypochlorite pentahydrate (NaClO, ≥ 39%) were supplied by Shanghai Aladdin Biochemical Technology Co, Ltd. Ruthenium(III) chloride hydrate (RuCl_3_·3H_2_O, 35.0-42.0% Ru basis) were bought from Macklin. All the chemicals and reagents were used as received without any further purification. The deionized (DI) water with a resistance of 18.2 MΩ·cm was obtained from the Milli-Q Plus System and used in the experiments.

**S2 Material characterization**

Scanning electron microscopy (SEM) images were acquired by employing a Zeiss Sigma 300 microscope housed in Jena, Germany. For transmission electron microscopy (TEM), an FEI Talos F200s was utilized. High-angle annular dark-field scanning transmission electron microscopy (HAADF-STEM) along with energy-dispersive X-ray spectroscopy (EDS) elemental mapping were carried out by means of the FEI Themis Z. X-ray powder diffraction (XRD) patterns were retrieved using a Rigaku Ultima IV system. The patterns were recorded within the 2θ range from 10° to 90° at a scanning speed of 5° min^−1^. As for X-ray photoelectron spectroscopy (XPS), it was executed using a Thermo Fisher Scientific K-Alpha + instrument. These advanced characterization techniques were employed to comprehensively analyze the samples, providing in-depth insights into their microstructure, elemental composition, and chemical states.

**S3 Preparation of the working electrode**

For the preparation of the working electrode, 4 mg of the catalysts were added to a mixture composed of 990 μL of ethanol and 10 μL of Nafion solution. This mixture was then subjected to ultrasonication for approximately 1 hour. The purpose of ultrasonication was to guarantee that the catalyst inks were well-dispersed. After achieving a homogeneous dispersion, the resulting catalyst inks were carefully pipetted and dropped onto two sheets of 1 cm^2^ carbon paper. This deposition process was carried out with precision to ensure an even distribution of the catalyst on the carbon paper surface, which is crucial for the subsequent electrochemical performance of the working electrode.

**S4 Quantification of ammonia**

The indophenol blue method was adopted to quantitatively measure the generated NH_3_. Standard NH_4_Cl solutions in various electrolytes (0.5 M K_2_SO_4_ diluted 1000-fold with 0.05 M H_2_SO_4_) were utilized to construct the calibration curves. In a typical experiment, 5 mL of standard NH_4_Cl solutions with different concentrations (0, 0.01, 0.02, 0.03, 0.05, 0.08, and 0.10 mM) were carefully pipetted into pre-cleaned vials. Subsequently, 2 mL of a 1 M NaOH solution containing 5 wt% salicylic acid and 5 wt% sodium citrate was added. Then, 1 mL of a 0.05 M NaClO solution and 0.2 mL of a 1 wt% sodium nitroferricyanide solution were introduced into the vials. After a reaction time of 2 hours, a UV-Vis Spectrophotometer was employed to obtain the UV-visible absorption spectra of the solutions. The absorbance at a wavelength of λ = 655 nm was used to measure the formed indophenol blue. To quantify the produced NH_3_, the same experimental procedure was followed, except that 0.05 mL of the post-electrolysis catholyte was used instead of the standard NH_4_Cl solutions. This approach ensured accurate determination of the ammonia content in the post-electrolysis samples, enabling reliable analysis of the electrocatalytic nitrate reduction process.

**S5 Quantification of nitrite**

A spectrophotometric approach was employed to quantify the concentration of nitrite (NO_2_^−^) in a solution. Initially, a color-developing reagent was prepared by mixing 4 g of p-amino benzene sulfonamide, 0.2 g of N-(1-Naphthyl) ethylenediamine dihydrochloride, 50 mL of ultrapure water, and 10 mL of phosphoric acid (with a density of 1.70 g/mL). For the analysis of the solution, a specific volume of the post-electrolysis catholyte was retrieved from the electrolytic cell. This catholyte was then diluted to a 5 mL solution with a suitable concentration. Next, 0.1 mL of the prepared color reagent was added to the 5 mL solution. The mixture was thoroughly agitated to ensure homogeneous distribution and then allowed to stand for 20 minutes. Afterward, a UV-Vis Spectrophotometer was utilized to acquire the UV-visible absorption spectra of the solution. The absorbance value at a wavelength of λ = 540 nm was subsequently used to calculate the concentration of nitrate in the solution. This method provides a reliable and accurate means of determining the nitrate concentration in the post-electrolysis catholyte samples.

**S6 Quantification of nitrate**

First, a certain quantity of the electrolyte was withdrawn from the electrolytic cell and subsequently diluted to a volume of 5 mL for the purpose of detection. Following this, 0.1 mL of a 1 M hydrochloric acid (HCl) solution and 0.01 mL of a 0.8 wt.% sulfamic acid solution were added to the diluted electrolyte solution. The resulting mixture was thoroughly stirred to ensure uniform mixing. Then, it was placed in a light-shielded environment and left undisturbed for 20 minutes. Next, the absorption spectra of the treated solution were measured by means of a UV-Vis spectrophotometer. During the measurement, the absorption intensities at wavelengths of 220 nm and 275 nm were carefully recorded. The final absorbance value was calculated according to the formula: A = A_220nm_ − 2A_275nm_. Finally, a series of standard potassium nitrate (KNO_3_) solutions were utilized to establish a calibration curve between concentration and absorbance. This calibration curve would serve as a reference for accurately determining the concentration of the analyte in the electrolyte samples based on their measured absorbance values.

**S7 Electrocatalytic measurements**

In this study, the electrocatalytic test was performed at ambient temperature in an H-type cell that was partitioned by an ion-exchange membrane, specifically Nafion 117. Prior to commencing the electrocatalytic test, the Nafion 117 membrane was subjected to a series of treatments. It was first soaked in a 5% H_2_O_2_ solution at 80 °C for approximately one hour. Subsequently, it was immersed in a 0.5 M H_2_SO_4_ solution at the same temperature (80 °C) for another hour. Finally, it was thoroughly rinsed with deionized water. An Ivium-n-Stat electrochemical workstation equipped with multiple channels was employed to acquire the electrochemical data. In a standard three-electrode setup, a platinum (Pt) plate functioned as the counter electrode, an Ag/AgCl electrode filled with a saturated potassium chloride (KCl) solution served as the reference electrode, and the catalyst supported on carbon paper acted as the working electrode. All the potentials measured during this work were initially referenced with respect to the Ag/AgCl electrode. They were then converted to the reversible hydrogen electrode (RHE) scale using the subsequent equation: E (vs RHE) = E (vs Ag/AgCl) + 0.197 V + 0.059 × pH. For the electrocatalytic reduction of nitrate, an electrolyte solution consisting of 0.5 M K_2_SO_4_ and 0.1 M KNO_3_ was utilized. Before conducting the test, the solution was purged with high-purity argon (Ar) gas for a minimum of 30 minutes to eliminate any dissolved oxygen. Subsequently, 40 mL of the electrolyte was separately introduced into the anode and cathode compartments of the H-type cell. A linear sweep voltammetry (LSV) curve was generated at a scan rate of 10 mV s^−1^, within a potential range spanning from 0.2 to −0.6 V (vs RHE). The chronoamperometry test was carried out for 1 hour at each potential, with a stirring rate maintained at 400 rpm. By comparing the LSV curves of RuM (M=Fe, Co, Ni, Cu)-NC in the presence and absence of KNO_3_ addition, distinct differences in current density were observed when the potential was more negative than −0.1 V (vs RHE). This observation clearly indicated that the nitrate reduction reaction occurred effectively. The potential range selected for the potentiostatic test was from −0.1 to −0.5 V (vs RHE). The process of refreshing the electrolyte and commencing the next cycle of electrolysis under identical conditions took approximately 3-5 minutes. CV curves were obtained within the non-Faradaic region at different scan rates, namely 20, 40, 60, 80, and 100 mV s^−1^, in order to determine the electrochemical double-layer capacitance (C_dl_).

**S8 Electrochemical active area normalization**

The formula for calculating the normalized current density of ECSA for the preparation of catalysts is:

ECSA-normalized current density = current density×C_s_ / C_dl_ (S1)

ECSA could be determined based on the equation of *ECSA* = *C_dl_/C_s_*, where *C_dl_* and *C_s_* refer to double-layer capacitance and specific capacitance, respectively. As the *C_s_* may change depending on the composition of the electrocatalyst, it is determined from independent experiment utilizing known-area electrode according to the following equation:

$\boldsymbol{C}_{\boldsymbol{s}}\boldsymbol{=}\frac{\int_{\boldsymbol{V}_{\boldsymbol{1}}}^{\boldsymbol{V}_{\boldsymbol{2}}} \boldsymbol{IdV}}{\boldsymbol{2}\boldsymbol{v}\boldsymbol{\Delta V}}$ (S2)

Where $\int_{V_{1}}^{V_{2}} \mathrm{IdV}$ is the area of CVs, *v* is scan rate (V s^-1^), and $\Delta V$ is potential window. Thus, *C_s_* is the slope of scan rate and $\frac{\int_{V_{1}}^{V_{2}} \mathrm{IdV}}{2\Delta V}$plot [S1].

**S9 XPS valence band spectrum calculations for d-bandcenter**

Firstly, the energy of the original spectrum was calibrated (with the Fermi energy level as the reference zero point), the characteristic interval of −2-10 eV was intercepted (the specific range needs to be adjusted according to the characteristics of the spectrum), and then the background signal was deducted by the Shirley method. Then, calculate the product integral of numerical integration (∫ N(ε)ε dε) and total intensity integral (∫ N(ε) dε) by photoelectron intensity (N(ε)) and energy (ε). Finally, the ratio of the two is taken as the center value of the D band (E_d_ = ∫ N(ε)ε dε / ∫ N(ε) dε). The physical basis of this method is derived from Hammer and Nørskov's d-band theory, in which the numerical position of the E_d_ directly reflects the orbital hybridization intensity of the transition metal surface state and the adsorbed molecule: when the E_d_ is large and negative (away from the Fermi level), the adsorption is weak, while when it is close to the Fermi level, it indicates strong chemisorption activity [S2-S4].

**S10 Electrochemical in situ ATR-SEIRAS reflection analysis**

In-situ attenuated total reflection reflectance surface-enhanced infrared spectroscopy (ATR-SEIRAS) represents a powerful method for probing the interaction occurring between the surface of a solid catalyst and a solution. First, a Si prism plated with gold and catalysts is integrated into a self-fabricated spectral-electrochemical cell. In this cell, a Pt wire functions as the counter electrode, while an Ag/AgCl electrode serves as the reference electrode. Subsequently, the assembled cell is firmly fixed within a self-constructed optics system that is installed inside the chamber of a Nicolet Is50 FTIR spectrometer. This setup is specifically designed for conducting electrochemical ATR-FTIR measurements. The reporting of ATR-FTIR spectra follows a similar approach to that of thin-layer in-situ FTIR. The sample spectra are systematically collected within the potential range spanning from 0 to −0.6 V.

**S11 Electrochemical in situ Raman spectroscopy**

A Lab-RAM HR Raman microscopy system (Horiba Jobin Yvon, HR550) was employed to carry out Raman spectroscopy. This system was outfitted with a 532 nm laser for excitation purposes, a water-immersion objective (Olympus LUMFL, 60×, numerical aperture = 1.10), a monochromator featuring an 1800 grooves/mm grating, and a Synapse CCD detector. Each Raman spectrum was obtained by averaging three consecutively acquired spectra. The collection time for each individual spectrum was set at 60 seconds. In the case of in-situ Raman tests, an electrochemical cell with a three-electrode setup was utilized. Here, Pt wires were used as the counter electrode, and an Ag/AgCl electrode served as the reference electrode. The supporting electrolytes commonly consisted of 0.5 M K_2_SO_4_, and the tests were also conducted in an environment where 0.1 M KNO_3_ was present.

**S12 In-situ EPR Spectroscopy measurement**

A Bruker EMX PLUS spectrometer was utilized to conduct an in-situ Electron Paramagnetic Resonance (EPR) measurement. An electrochemical cell with a three-electrode configuration was used. Specifically, Pt wires functioned as the counter electrode, while an Ag/AgCl electrode served as the reference electrode. NO_3_RR was performed in an electrolyte composed of 0.5 M K_2_SO_4_. The reaction was carried out at a potential of −1.01 V relative to RHE, and it was investigated both when 0.1 M KNO_3_ was present in the electrolyte and when it was absent. In order to detect hydrogen radicals, 40 μL of the catholyte was extracted and combined with 40 μL of a 0.2 M aqueous solution of 5,5-dimethyl-1-pyrroline-N-oxide (DMPO). Ultimately, the EPR signals from these mixtures were gathered.

**Supplementary Figures and Tables**


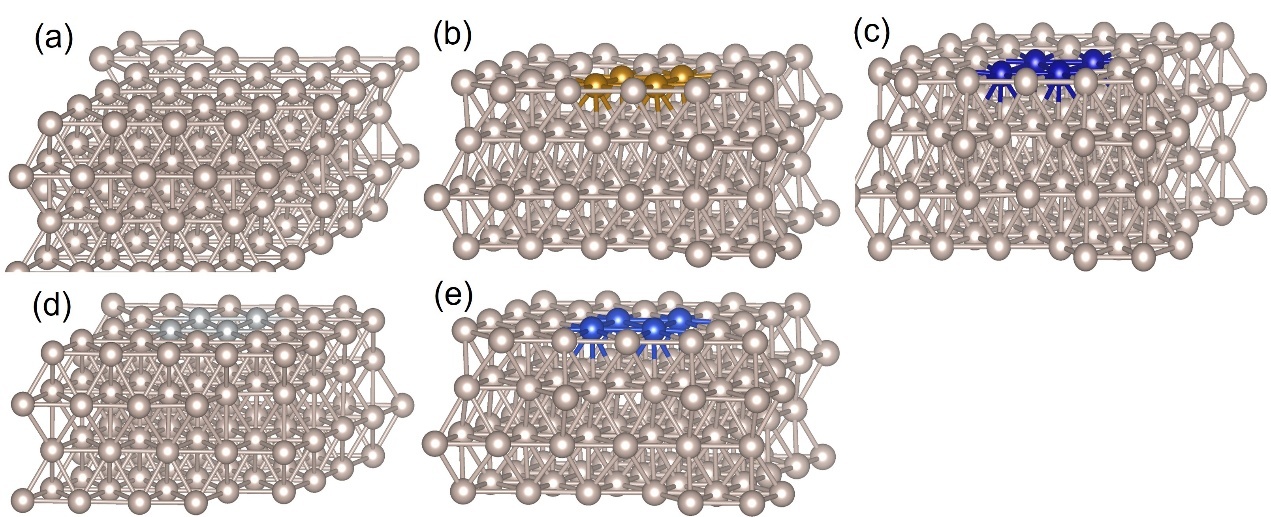


**Fig. S1** The optimized configuration for **a** Ru-NC, **b** RuFe_4_-NC, **c** RuCo_4_-NC, **d** RuNi_4_-NC and **e** RuCu_4_-NC doped with four atoms

**
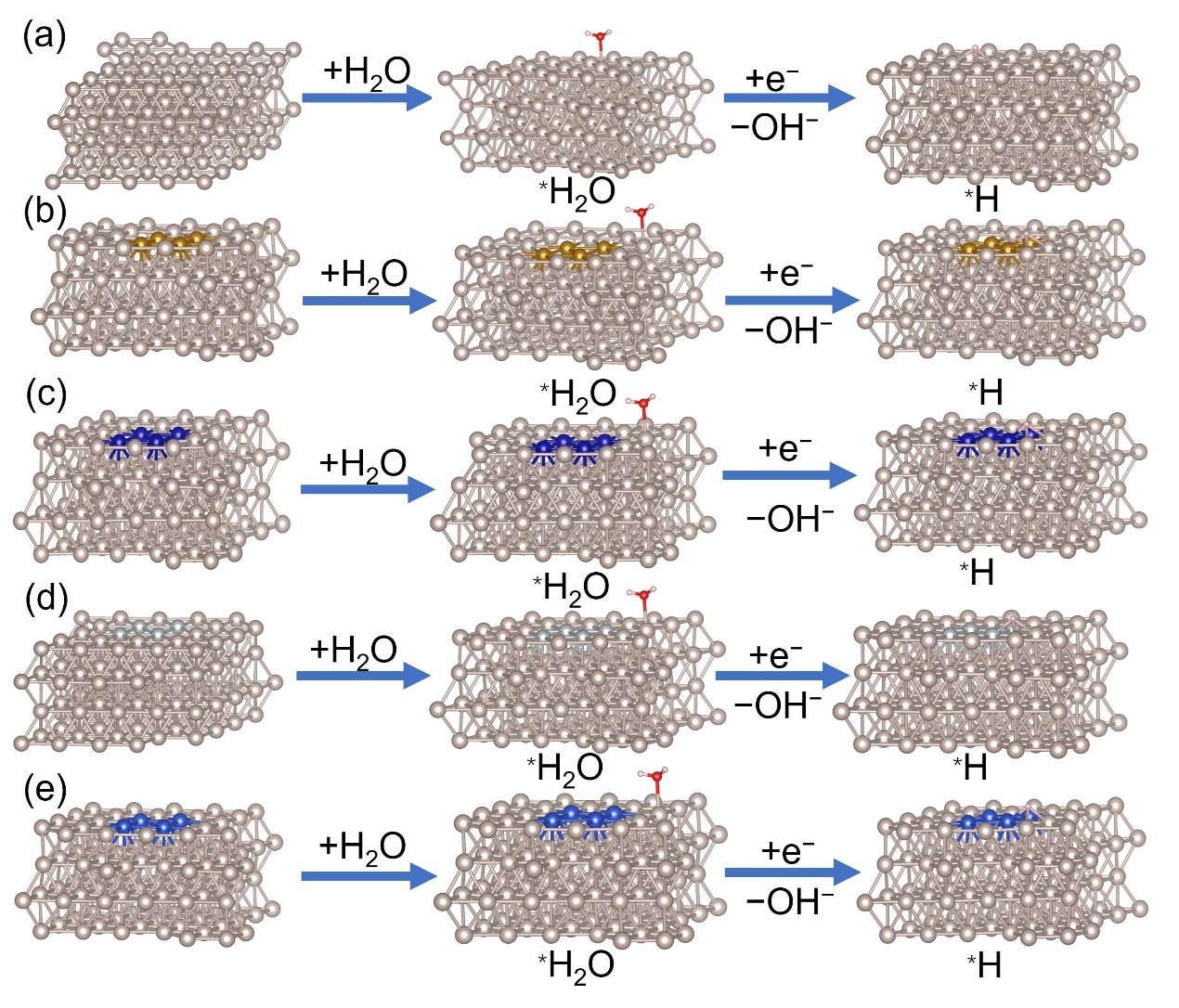
**

**Fig. S2** The optimized adsorption configuration for hydrogen evolution reaction (HER) on **a** Ru-NC, **b** RuFe_4_-NC, **c** RuCo_4_-NC, **d** RuNi_4_-NC and **e** RuCu_4_-NC doped with four atoms


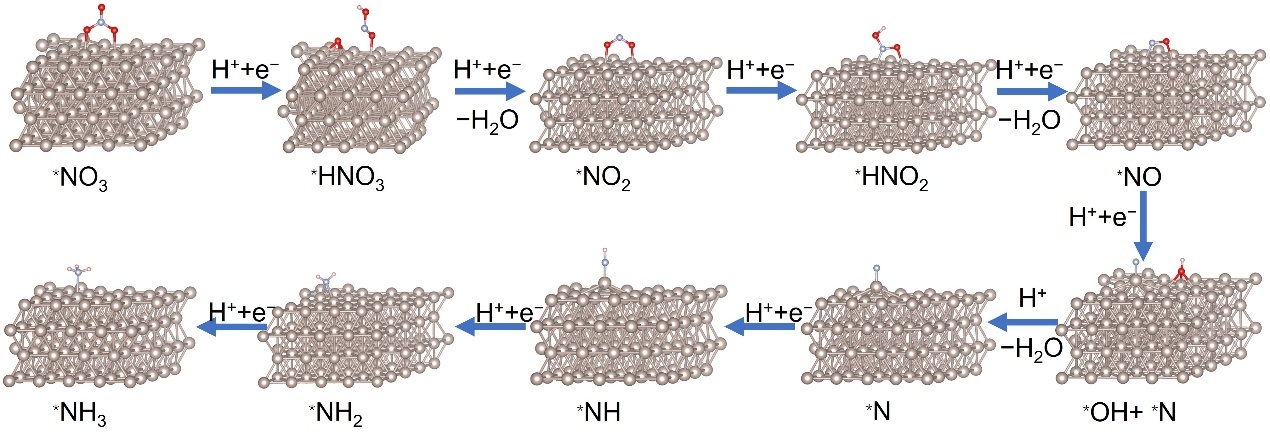


**Fig. S3** The optimized adsorption configuration for NO_3_RR to produce NH_3_ on Ru-NC


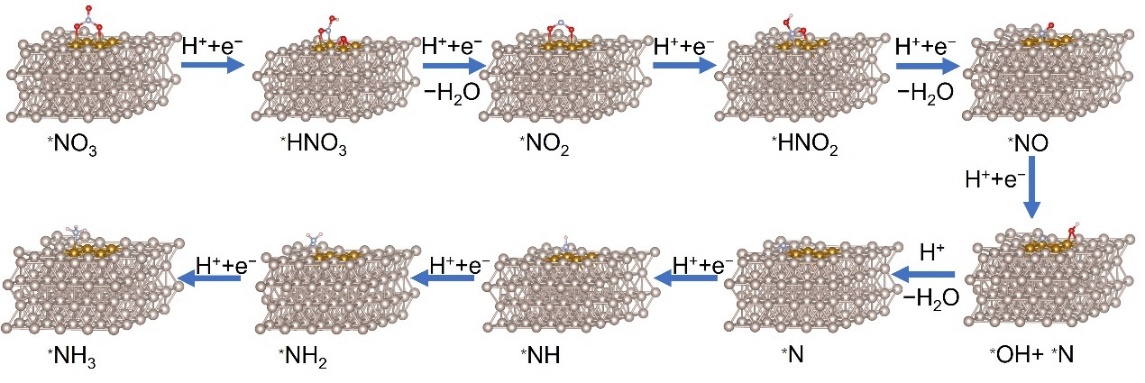


**Fig. S4** The optimized adsorption configuration for NO_3_RR to produce NH_3_ on RuFe_4_-NC doped with four atoms


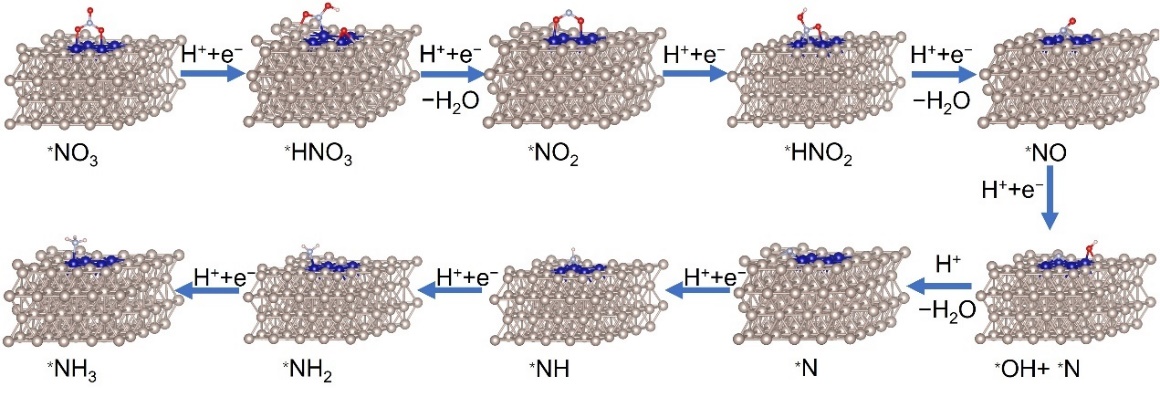


**Fig. S5** The optimized adsorption configuration for NO_3_RR to produce NH_3_ on RuCo_4_-NC doped with four atoms


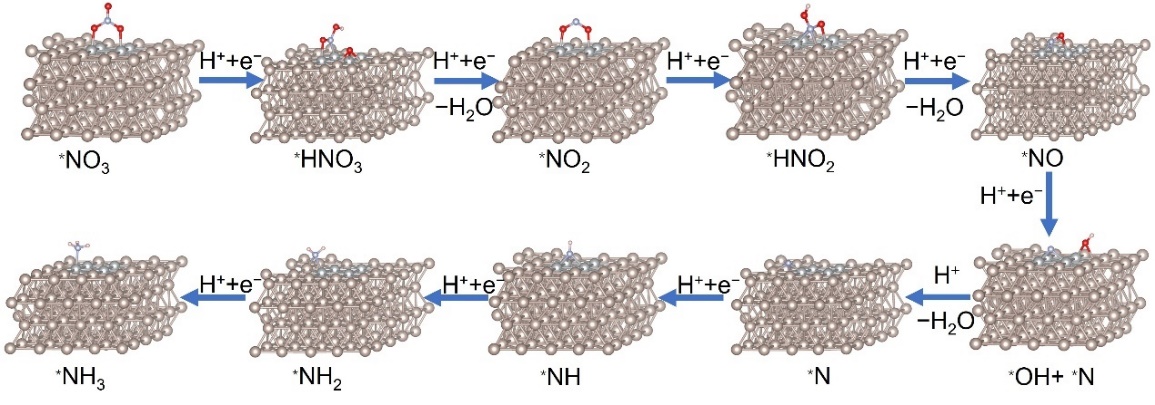


**Fig. S6** The optimized adsorption configuration for NO_3_RR to produce NH_3_ on RuNi_4_-NC doped with four atoms


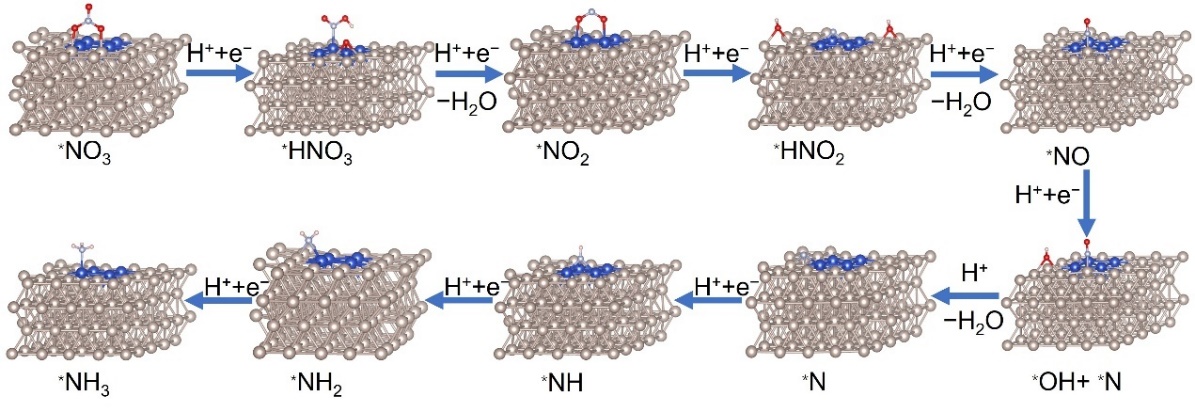


**Fig. S7** The optimized adsorption configuration for NO_3_RR to produce NH_3_ on RuCu_4_-NC doped with four atoms

**
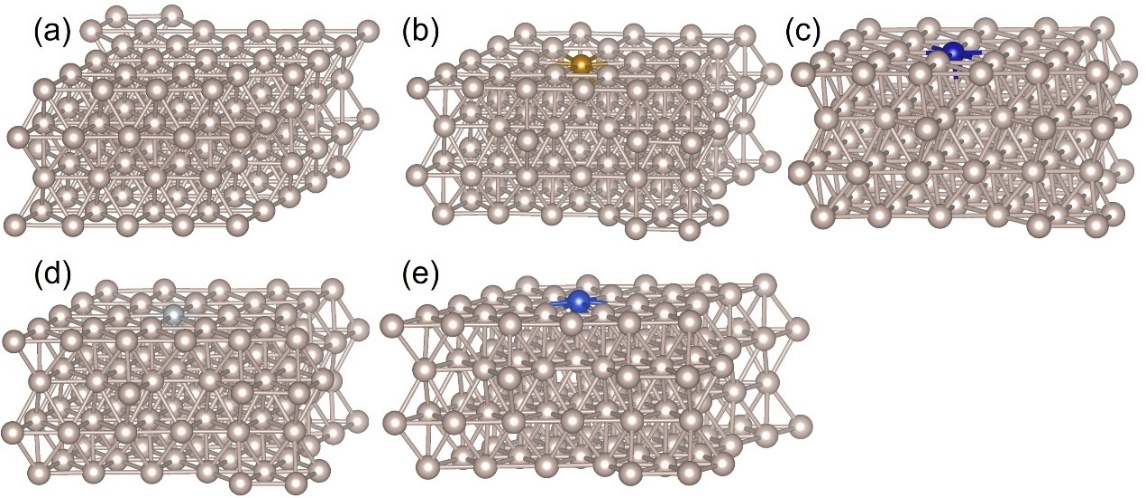
**

**Fig. S8** The optimized configuration for **a** Ru-NC, **b** RuFe-NC, **c** RuCo-NC, **d** RuNi-NC and **e** RuCu-NC doped with one atom

**
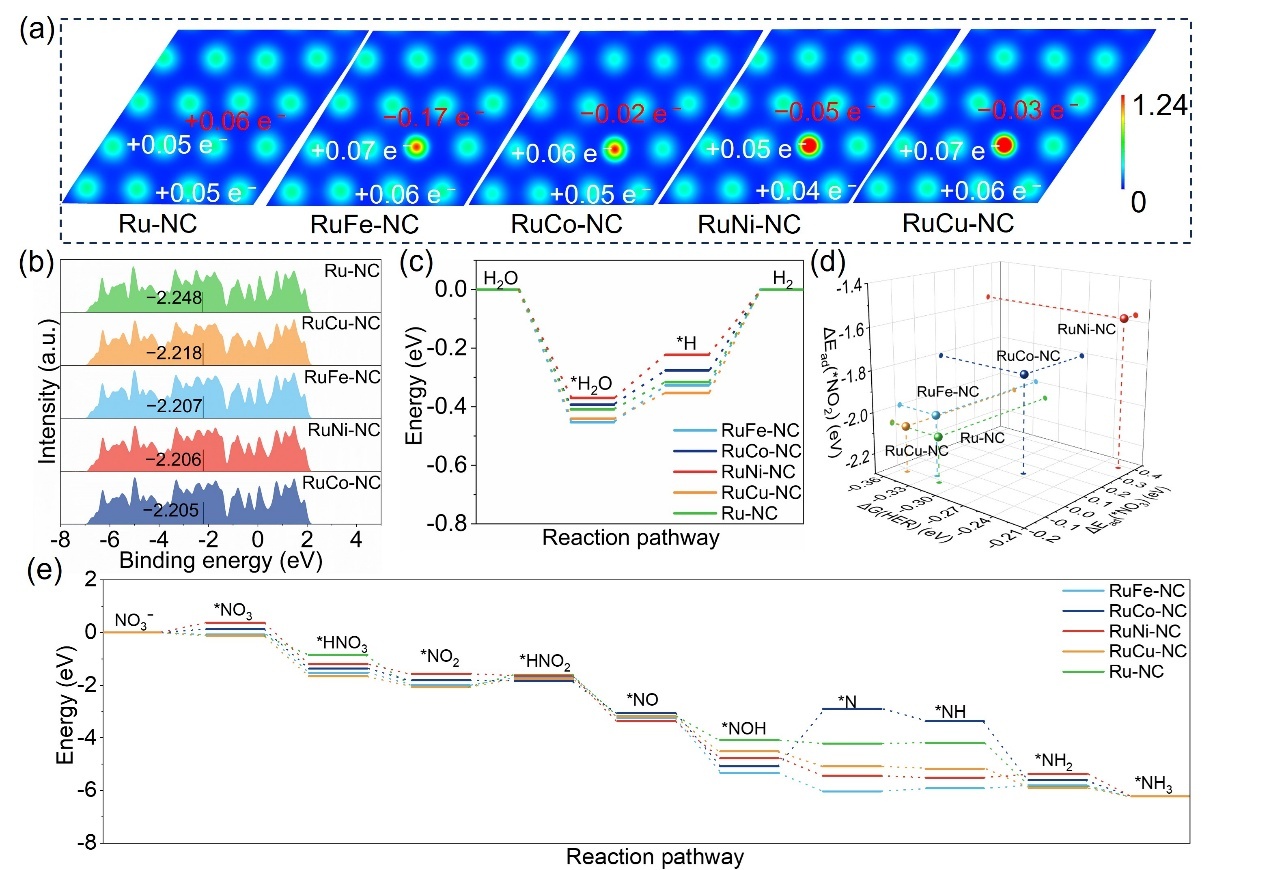
Fig. S9** Theoretical simulations of electrochemical NO_3_RR. **a** 2D slice passing through the first layer atoms on the Fe, Co, Ni and Cu-doped Ru surface. The is surface value in a is 0.1e^−^/Bohr^3^. Red and blue in the charge density difference plot represent electron accumulation and depletion. **b** d-band center positions of the RuM-NC nanoalloys. **c** The reaction energy changes of HER on the Ru-M site and pure Ru site. **d** The adsorption energy of *NO_3_, *NO_2_, and Gibbs free energy for H_2_ formation on Ru with various transition metal dopants. **e** Reaction free energy diagrams of NO_3_^−^-to-NH_3_


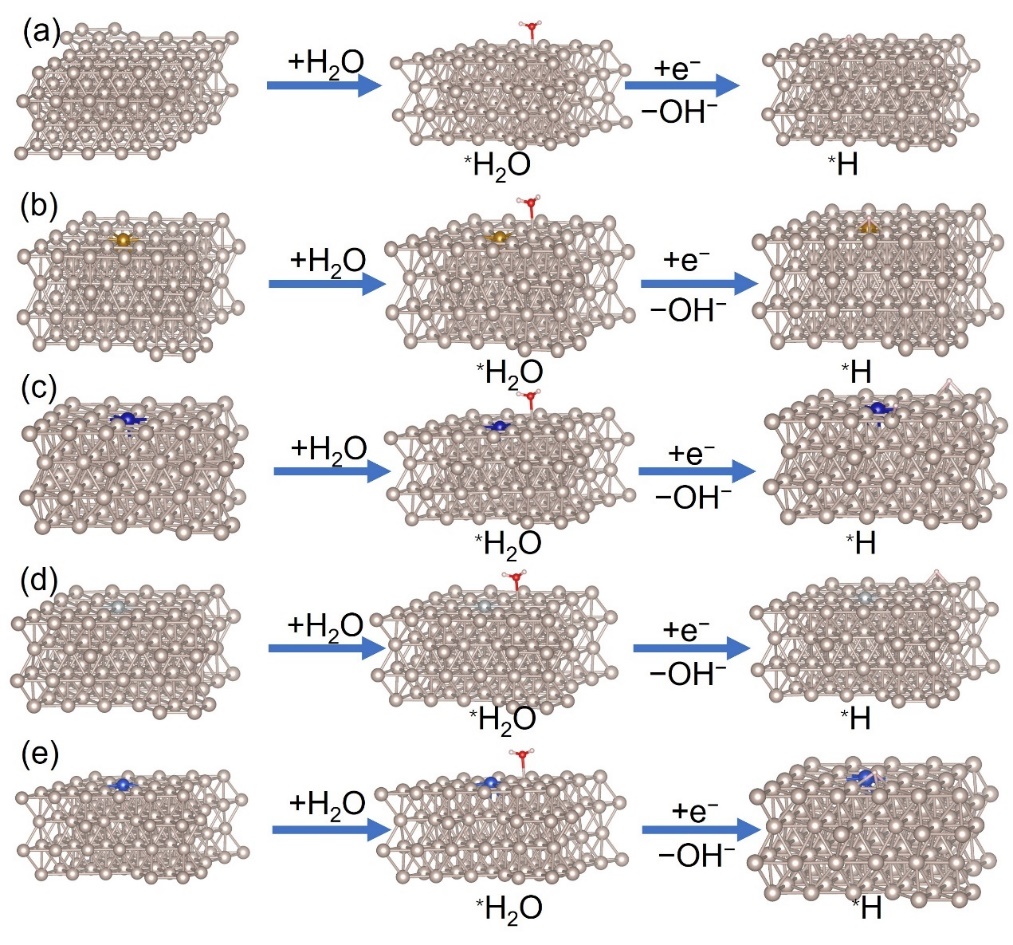


**Fig. S10** The optimized adsorption configuration for HER on **a** Ru-NC, **b** RuFe-NC, **c** RuCo-NC, **d** RuNi-NC and **e** RuCu-NC doped with one atom


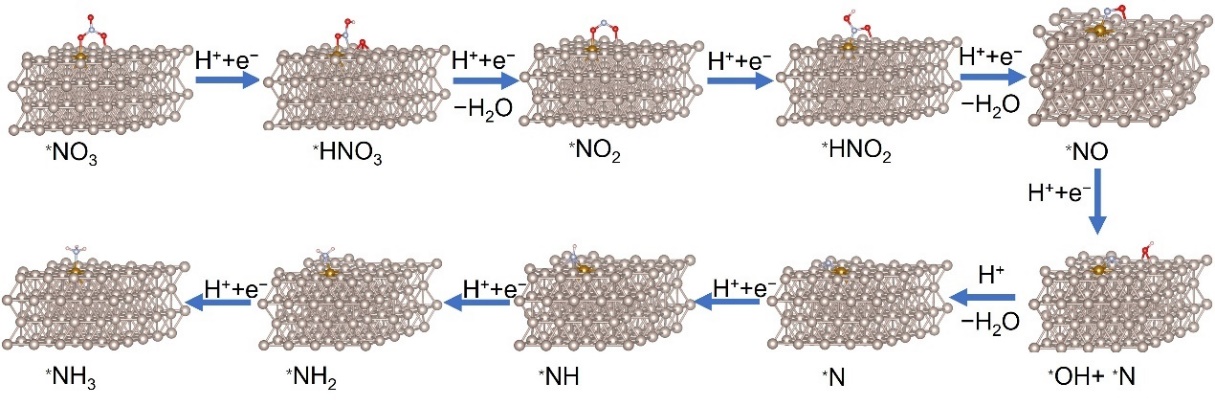


**Fig. S11** The optimized adsorption configuration for NO_3_RR to produce NH_3_ on RuFe-NC doped with one atom


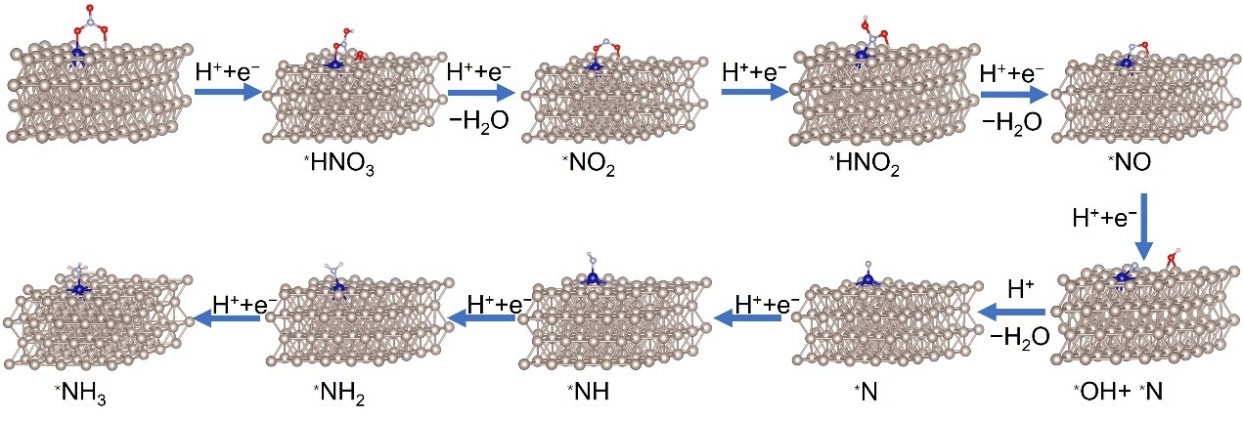


**Fig. S12** The optimized adsorption configuration for NO_3_RR to produce NH_3_ on RuCo-NC doped with one atom


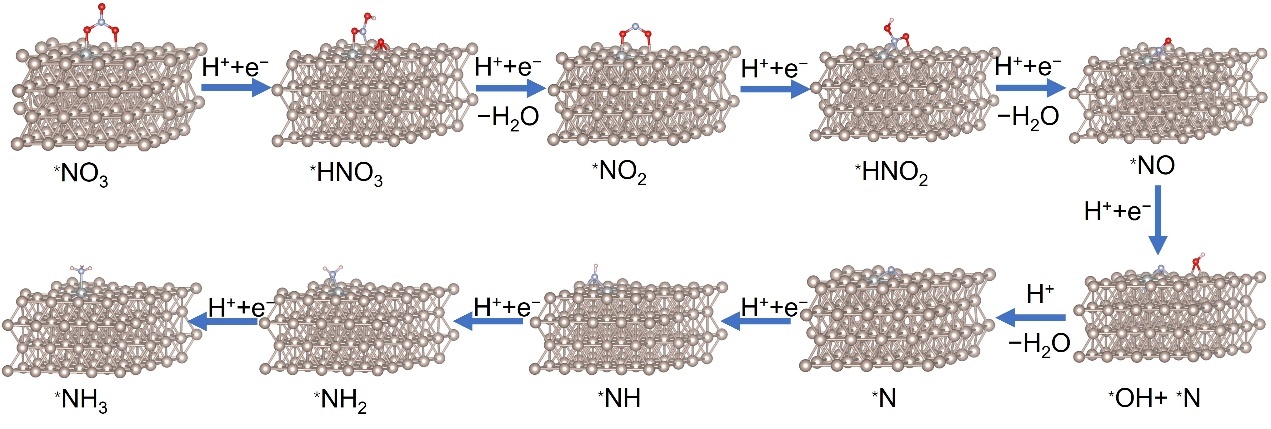


**Fig. S13** The optimized adsorption configuration for NO_3_RR to produce NH_3_ on RuNi-NC doped with one atom


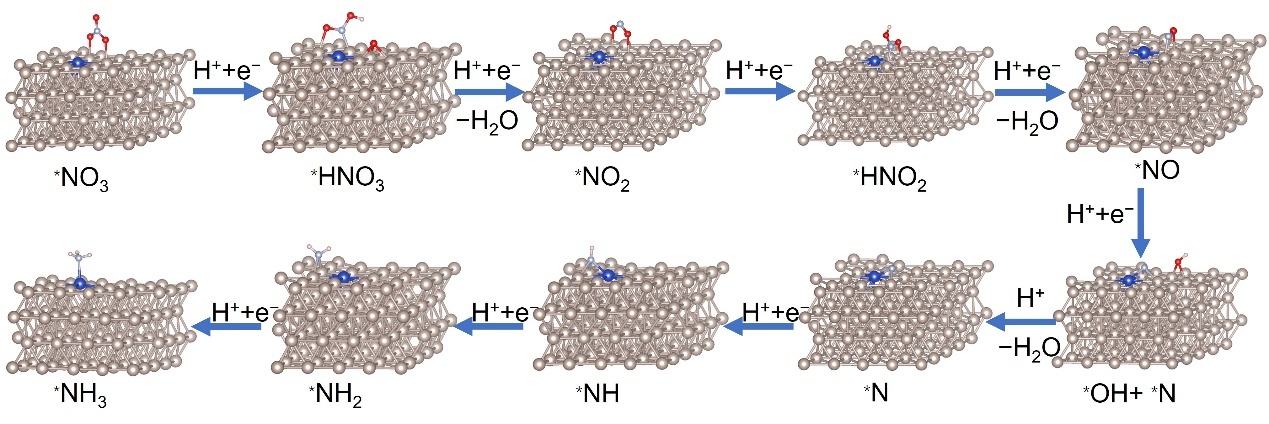


**Fig. S14** The optimized adsorption configuration for NO_3_RR to produce NH_3_ on RuCu-NC doped with one atom

**Supplementary Text S1**

The structural models, comprising Ru(101) surfaces with transition metal doped, reveal significant electronic redistribution at Ru-M interfaces (Fig. S8). As illustrated in Figs. S9a and 1a, a single doped transition metal atoms M in the Ru (101) plane depletes more electrons compared to sites with four doped transition metal atoms M. This indicates that the number of doped atoms can effectively modulate the electronic distribution of the transition metal atom M and its adjacent Ru sites (Figs. 1b, S9b). The free energy changes for the conversion of *H_2_O to *H follows the order: RuNi-NC (0.147 eV) > RuFe-NC (0.126 eV) > RuCo-NC (0.117 eV) > Ru-NC (0.093 eV) > RuCu-NC (0.088 eV). Additionally, the energy required for H_2_ production from *H ranks as follows: RuCu-NC (0.353 eV) > RuFe-NC (0.327 eV) > Ru-NC (0.316 eV) > RuCo-NC (0.276 eV) > RuNi-NC (0.223 eV) (Figs. S10, S9c). These results suggest that the Ru-Cu site inhibits the transition from *H to H_2_ to achieve the purpose of inhibiting HER, while the Ru-Fe, Ru-Co, and Ru-Ni sites inhibit HER by inhibiting the formation of *H. In conclusion, doping with transition metals has a positive effect on the inhibition of HER (Figs. S9a, c).

The calculated d-band centers for RuFe-NC, RuCo-NC, RuNi-NC, RuCu-NC, and Ru-NC are −2.207, −2.205, −2.206, −2.218, and −2.248 eV, respectively (Fig. S9b). This implies that doping with transition metals shifts the d-band center closer to the Fermi level, thereby facilitating electron migration during the catalytic process. From the schematic diagram of adsorption energies for RuM-NC, it is evident that doping with Co and Fe results in moderate *NO_3_ and *NO_2_ adsorption energies while simultaneously inhibiting HER, making them promising candidates (Fig. S9d). These results suggest that the doped transition metal atoms in RuM nanoalloys alters their surface electronic states, potentially enhancing NO_3_RR performance.

Fig. S9e reveals that the Ru-M sites exhibit lower energy barriers of approximately 0.284, −0.023, −0.071, and 0.31 eV, respectively, compared to the 0.464 eV barrier of the Ru site (Figs. S3, S11-S14). These barriers correspond to the rate-limiting steps of *NO_2_ to *HNO_2_, demonstrating the synergic effect of the Ru−M sites in reducing the energy barrier for NO_3_RR to produce NH_3_. Moreover, in contrast to the Ru sites, the Ru-M sites facilitate a more spontaneous process from *NO to NH_3_. In addition, the Ru-Co site, although it has a larger energy barrier between *NOH and *N, has a higher overall spontaneity than Ru-NC (Fig. S9d). This indicates that the redistribution of electrons in RuM-NC enhances electron transfer and lowers the energy barrier for the *NO_2_ → *HNO_2_. In summary, these results demonstrate that the synergic effect of RuM-NC nanoalloys promotes *H formation on Ru sites and accelerates the kinetics of NH_3_ production on M sites, indicating the critical role of transition metal doping in optimizing NO_3_RR performance.


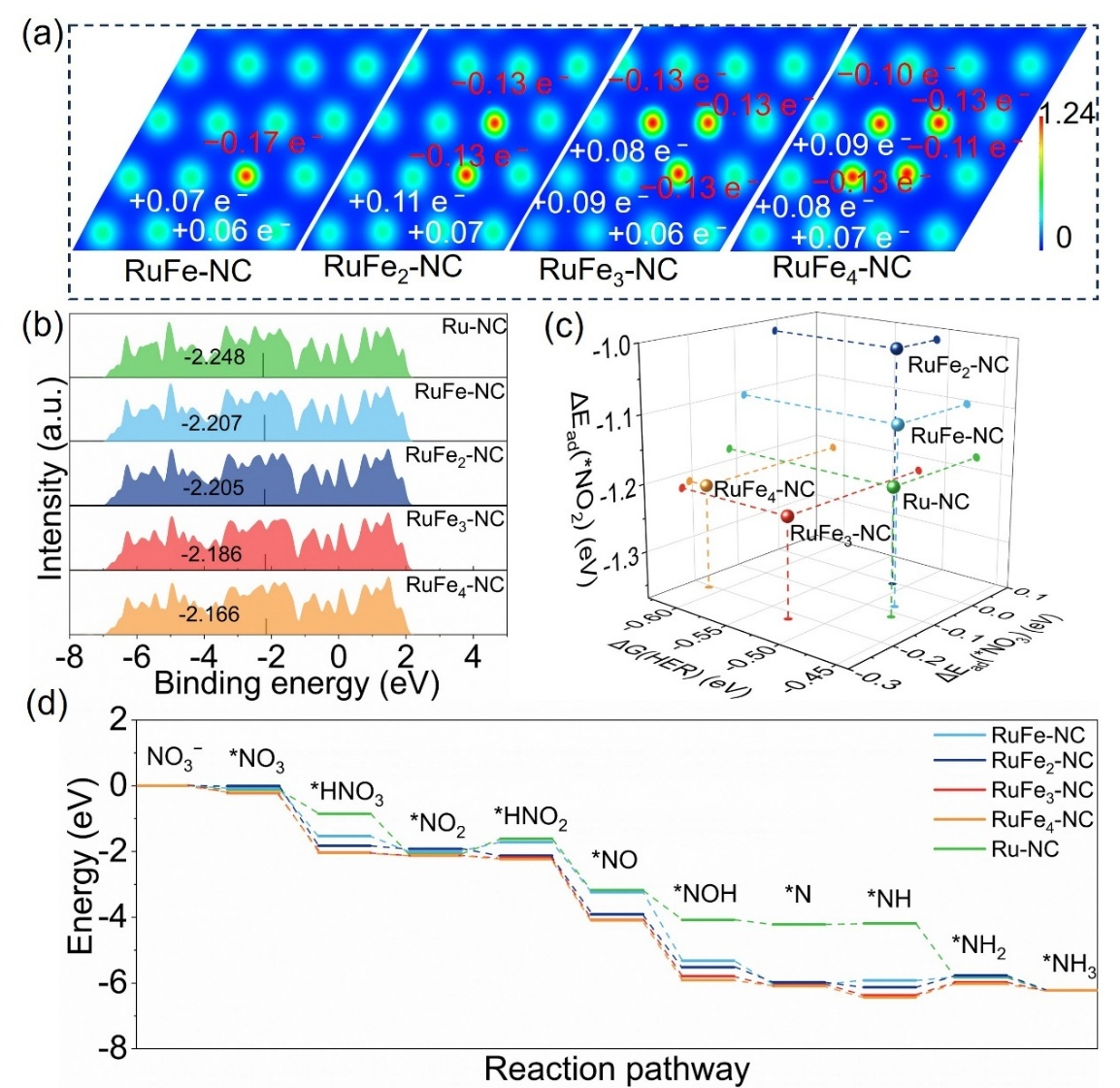


**Fig. S15 a** 2D slice passing through the first layer atoms on the Fe doped Ru surface. The is surface value in a is 0.1e^−^/Bohr^3^. Red and blue in the charge density difference plot represent electron accumulation and depletion. **b** d-band center positions of the RuFe_x_-NC (x=1,2,3,4). **c** The adsorption energy of *NO_3_, *NO_2_, and Gibbs free energy for H_2_ formation on Ru with various transition metal dopants. **d** Reaction free energy diagrams of NO_3_^−^-to-NH_3_


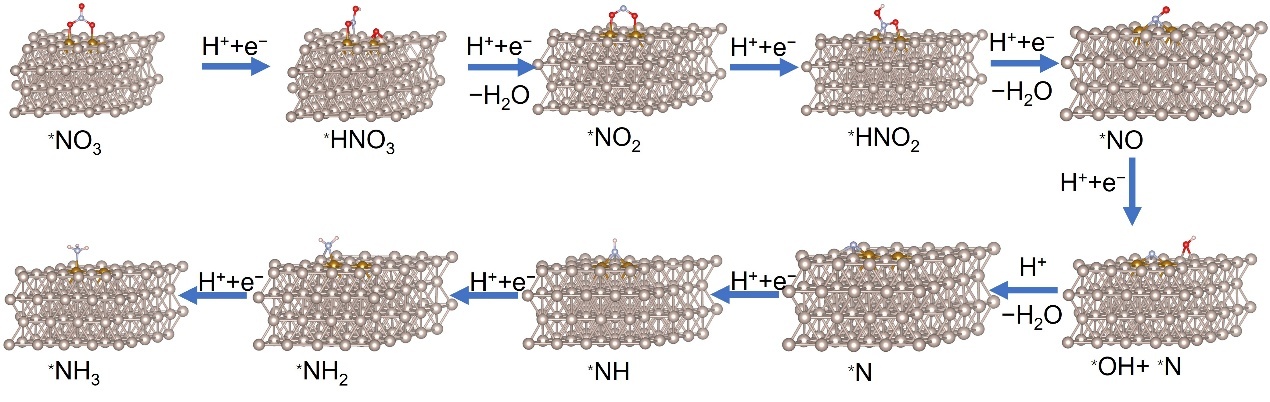


**Fig. S16** The optimized adsorption configuration for NO_3_RR to produce NH_3_ on RuFe_2_-NC doped with two atoms


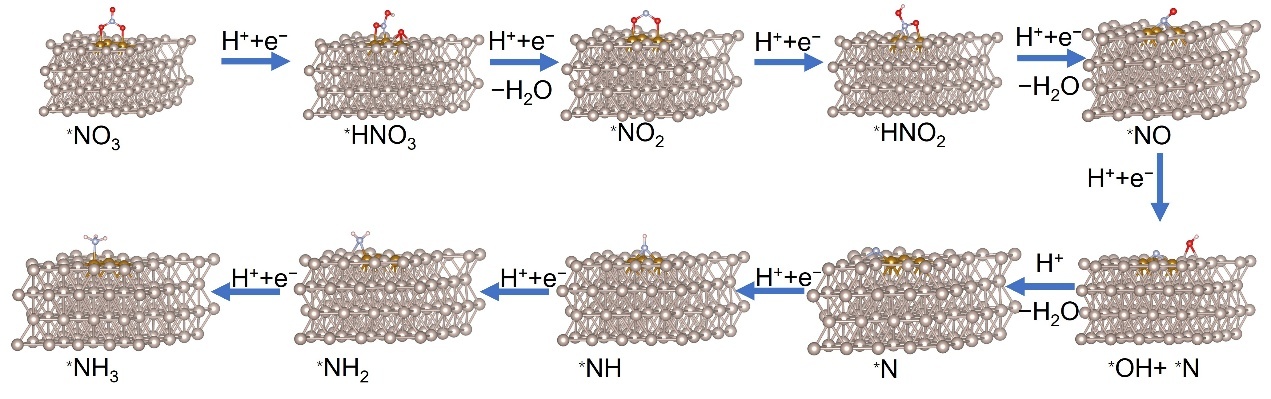


**Fig. S17** The optimized adsorption configuration for NO_3_RR to produce NH_3_ on RuFe_3_-NC doped with three atoms

**Supplementary Text S2**

In order to further confirm the effect of changing the number of doped transition metals on the electronic distribution of M and Ru sites, we continued to analyze RuFe-NC doped with one to four doped ones. As shown in Fig. S15a, as the number of doped atoms decreases, more electrons accumulate at the Fe site in Ru (101), suggesting that the number of doped atoms can modulate the electronic distribution of the transition metal atom M and its nearby Ru sites, agreeing to the d-band center shift (Figs. S15b, 2h). From the schematic diagram of the adsorption energy of Ru-M-NC, it can be determined that in these transition metals, doping four Fe will result in modest adsorption energies of *NO_3_ and *NO_2_ while inhibiting HER and may be promising candidates (Fig. S15c). Reaction free energy indicates that the Ru−Fe_x_ (x=1, 2, 3, 4) site exhibits lower barrier energy around 0.284, -0.212, -0.066, and -0.121eV than the 0.464 eV of the Ru site (Figs. S15d, S6, S11, S16 and S17), respectively, corresponding to the rate limiting-step of *NO_2_ to*HNO_2_, demonstrating the synergic effect of the Ru−Fe_x_ site lowering the energy barrier for NO_3_RR to produce NH_3_.


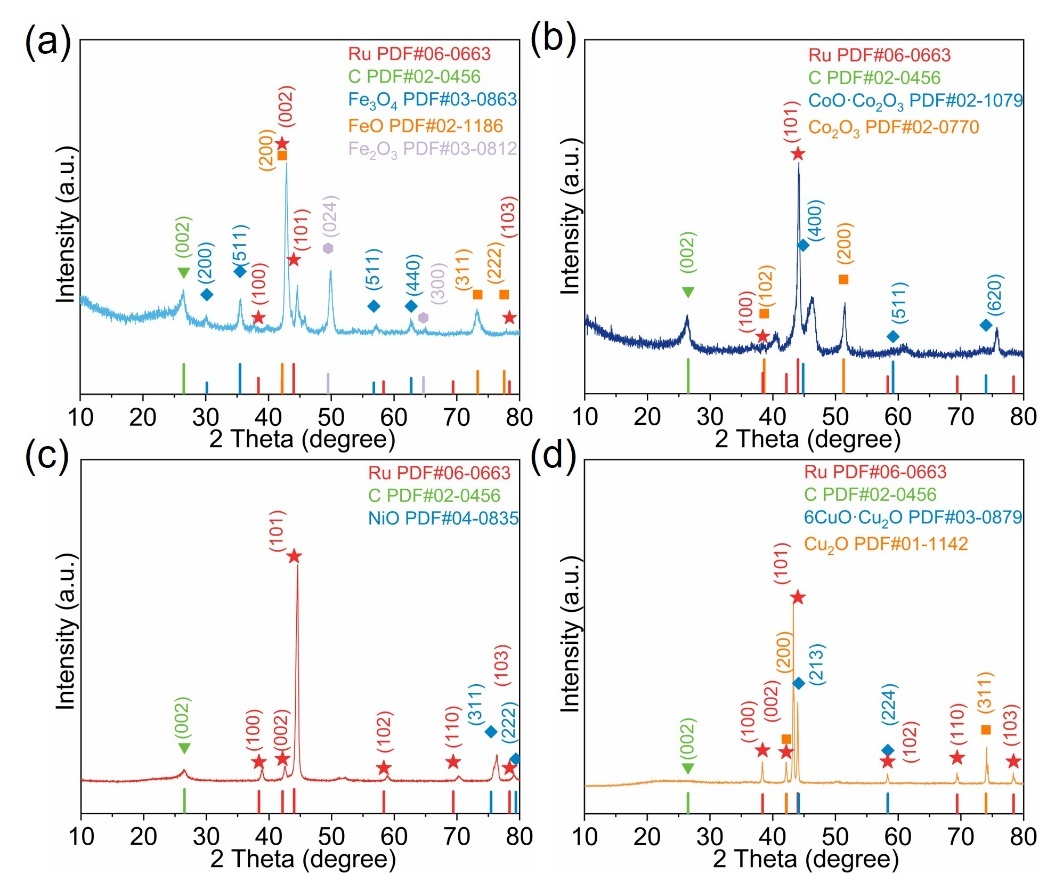


**Fig. S18** X-ray diffraction (XRD) pattern of the pyrolyzed obtained samples of **a** RuFe-NC, **b** RuCo-NC, **c** RuNi-NC, and **d** RuCu-NC


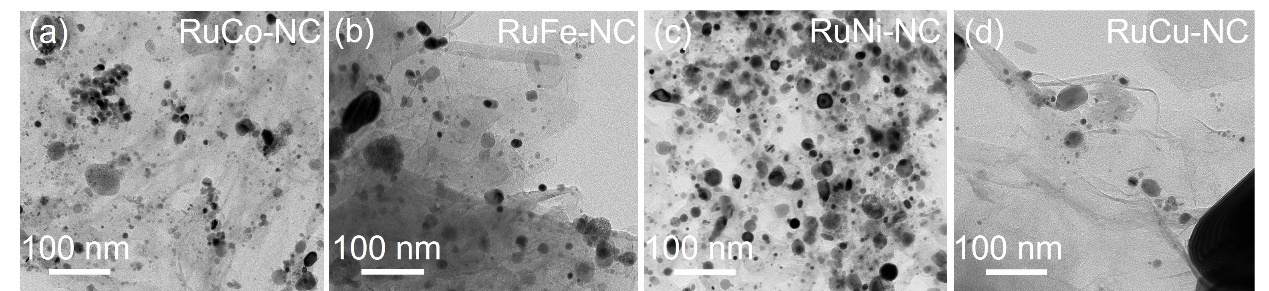


**Fig. S19** Transmission electron microscopy (TEM) of **a** RuCo-NC, **b** RuFe-NC, **c** RuNi-NC, and **d** RuCu-NC


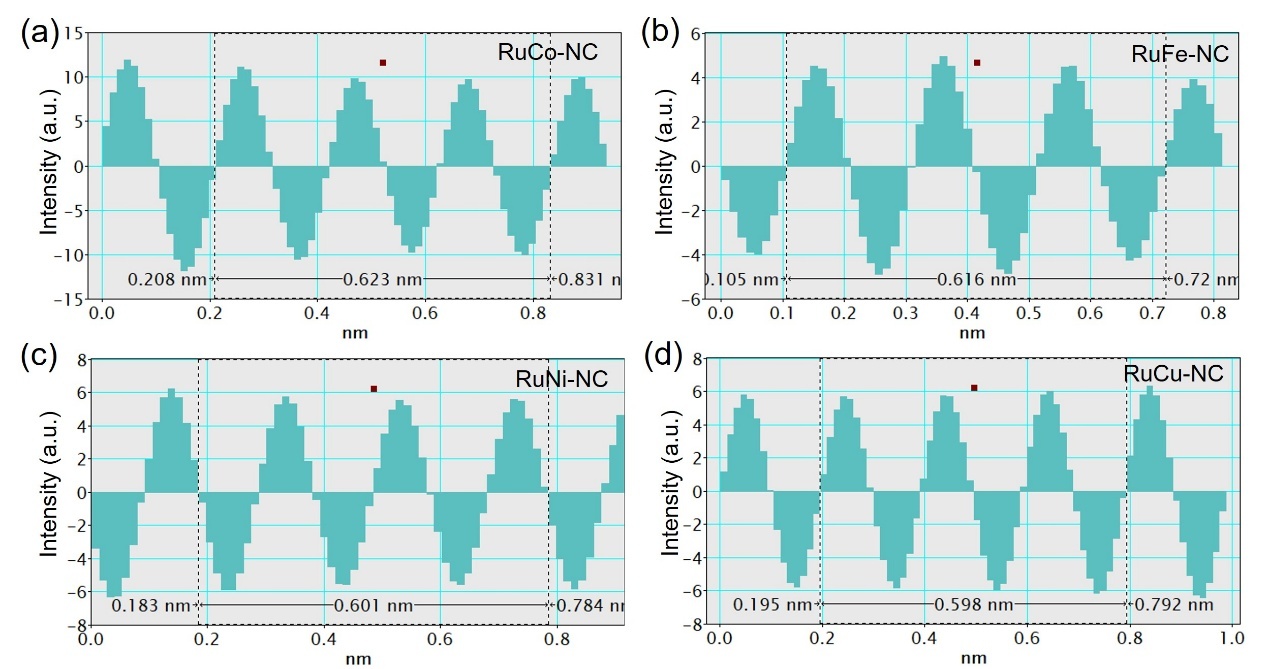


**Fig. S20** The lattice spacing of Ru (101) in **a** RuCo-NC, **b** RuFe-NC, **c** RuNi-NC, and **d** RuCu-NC

**Supplementary Text S3**

The X-ray diffraction (XRD) spectra of the a, b, c, d pyrolysis samples in Fig. S18 show typical peaks for iron (Fe) oxide (PDF #03-0863, #02-1186 and #03-0812), cobalt (Co) oxide (PDF #02-1079 and #02-0770), nickel (Ni) oxide (PDF #04-0835), and copper (Cu) oxide (PDF #03-0879 and #01-1142), respectively #21-0569), and both show carbon c (PDF #02-0456) and ruthenium metal (Ru) (PDF #06-0663). It was confirmed that M remained in the oxide state, while Ru was mainly in the metallic state, suggesting that chloride ions could selectively induce the formation of oxidized M. The induced effect of chloride ions for the formation of transition metals oxide was revealed. After the acid-etching treatment of the pyrolyzed samples, the morphology and crystalline structure of the RuM nanoalloys were further characterized. Notably, no nanoparticles of M oxides or metallic M were observed in the RuM samples (Fig. S19). Instead, Ru nanoparticles with an average size of approximately 2 nm were identified in RuFe-NC, RuCo-NC, RuNi-NC, and RuCu-NC, as evidenced by the lattice fringes corresponding to the Ru (101) plane (Figs. 2b-e and S20) [S5]. These results indicate that the acid-etching process selectively removes metals oxide nanoparticles while preserving the Ru nanoparticles, indicating the effectiveness of the etching strategy in tailoring the catalyst's structural properties.


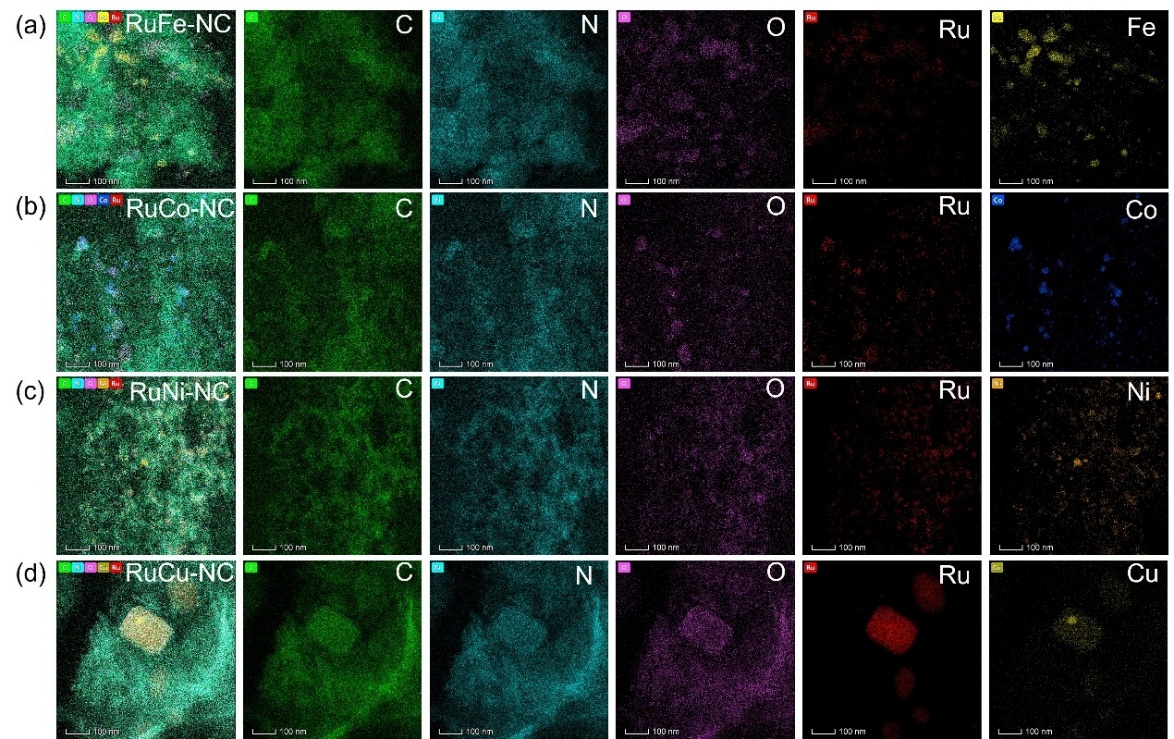


**Fig. S21** The energy-dispersive X-ray spectroscopy (EDS) element mapping images of **a** RuFe-NC, **b** RuCo-NC, **c** RuNi-NC and **d** RuCu-NC


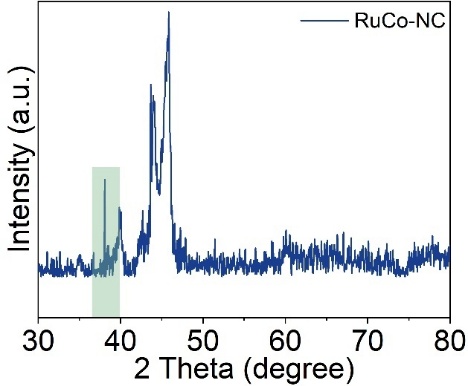


**Fig. S22** Raw X-ray diffraction pattern of RuCo


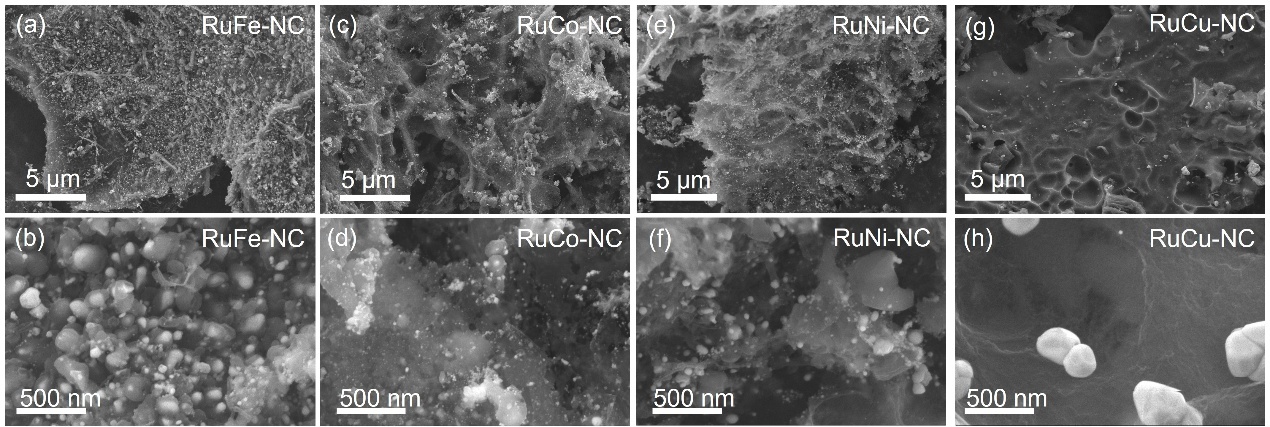


**Fig. S23** Scanning electron microscopy (SEM) images of **a, b** RuFe-NC, **c, d** RuCo-NC, **e, f** RuNi-NC and **g, h** RuCu-NC


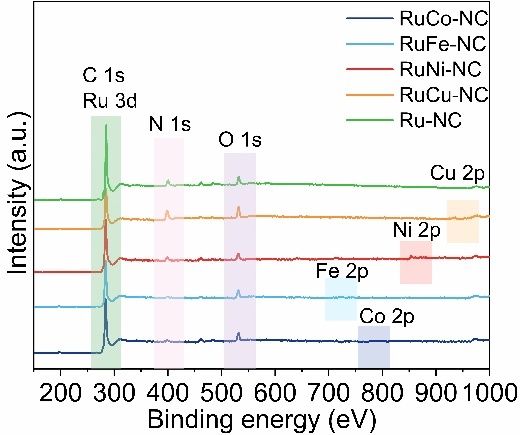


**Fig. S24** XPS survey of RuFe-NC, RuCo-NC, RuNi-NC, RuCu-NC and Ru-NC


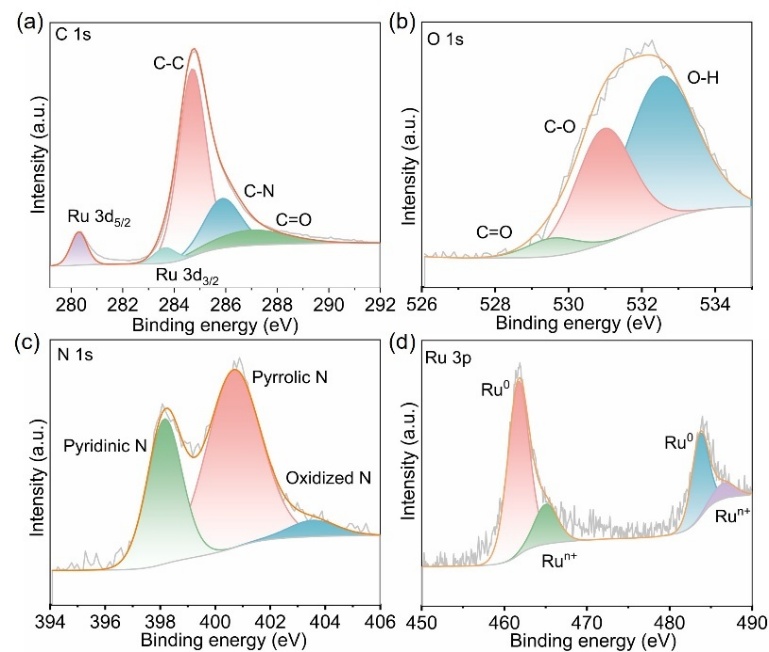


**Fig. S25** High-resolution XPS spectra of Ru-NC: **a** C 1s, **b** O 1s, **c** N 1s and **d** Ru 3p


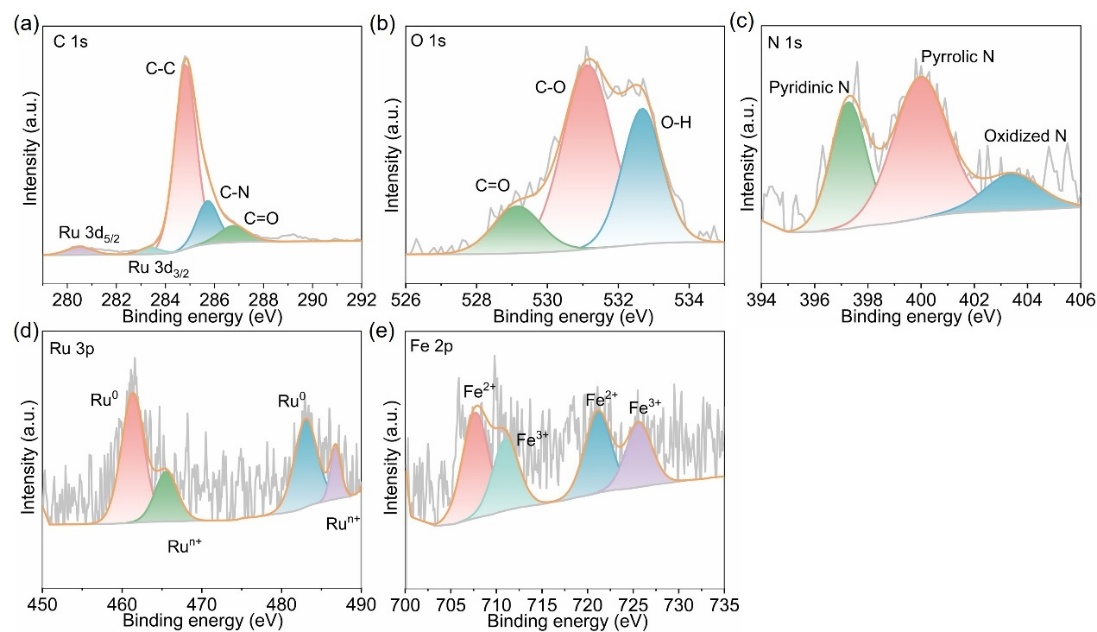


**Fig. S26** High-resolution XPS spectra of RuFe-NC: **a** C 1s, **b** O 1s, **c** N 1s, **d** Ru 3p, and **e** Fe 2p


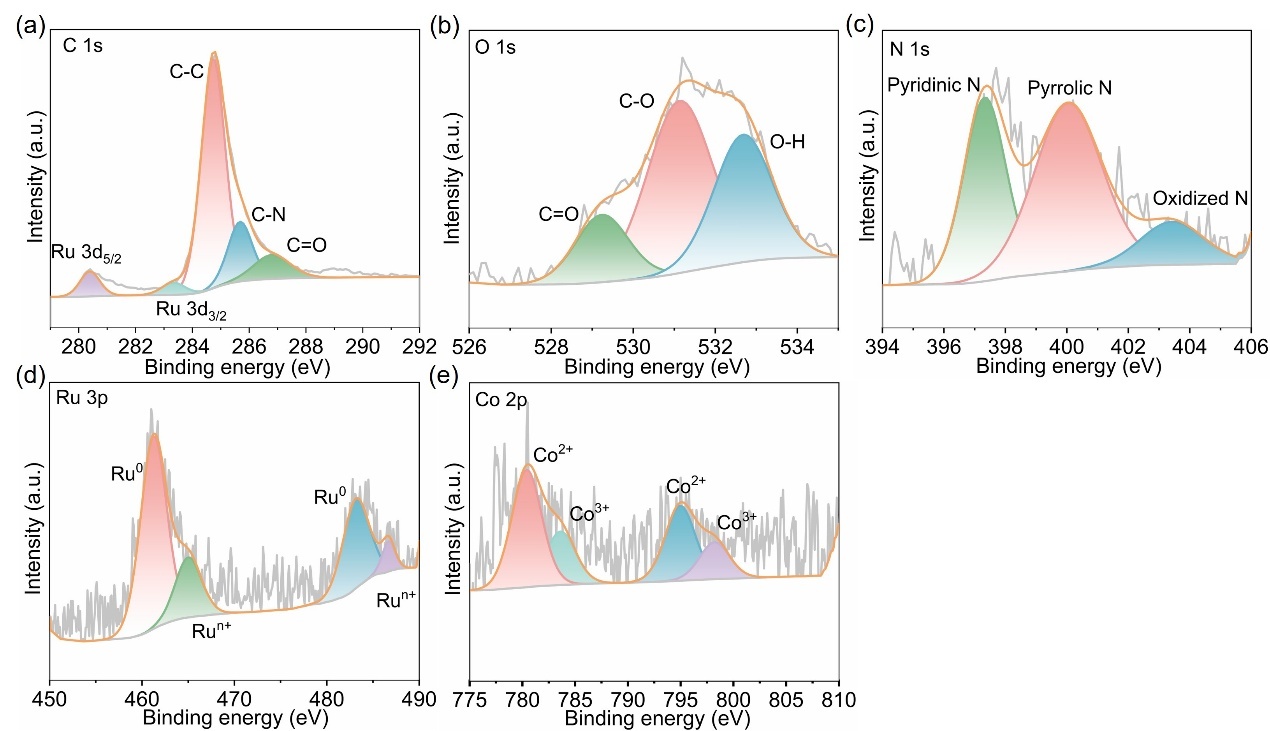


**Fig. S27** High-resolution XPS spectra of RuCo-NC: **a** C 1s, **b** O 1s, **c** N 1s, **d** Ru 3p, and **e** Co 2p


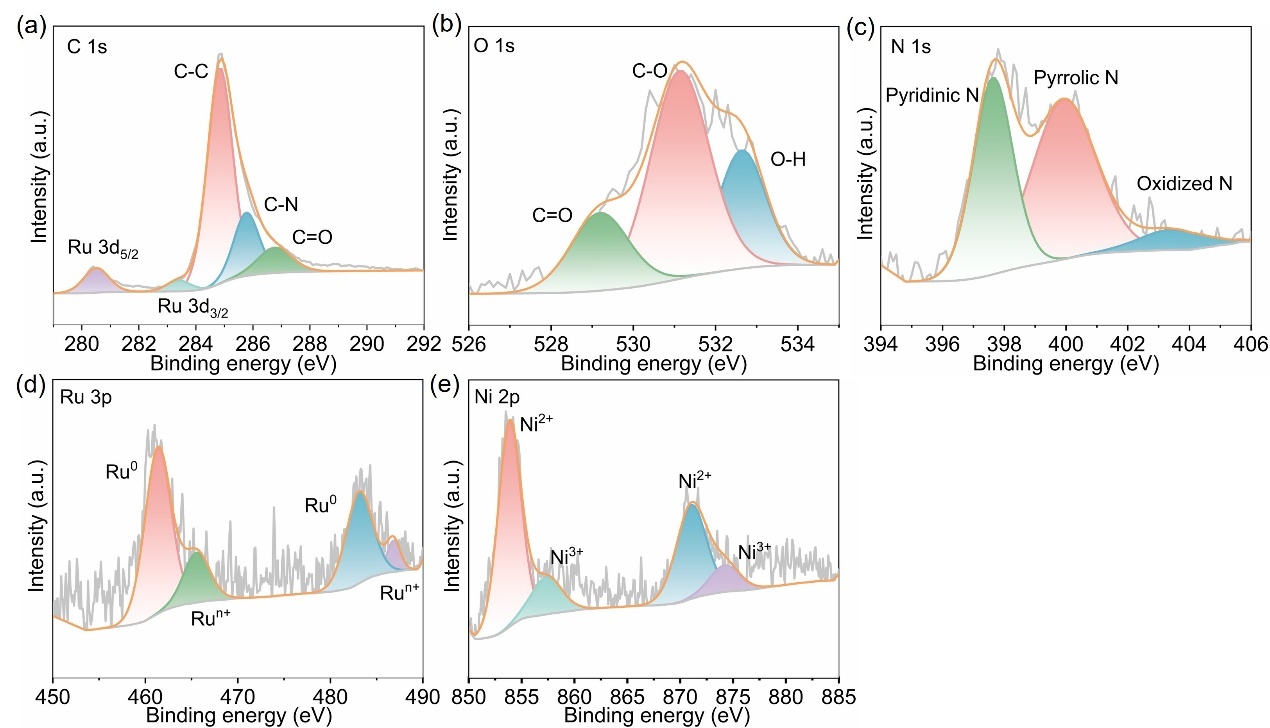


**Fig. S28** High-resolution XPS spectra of RuNi-NC: **a** C 1s, **b** O 1s, **c** N 1s, **d** Ru 3p, and **e** Ni 2p


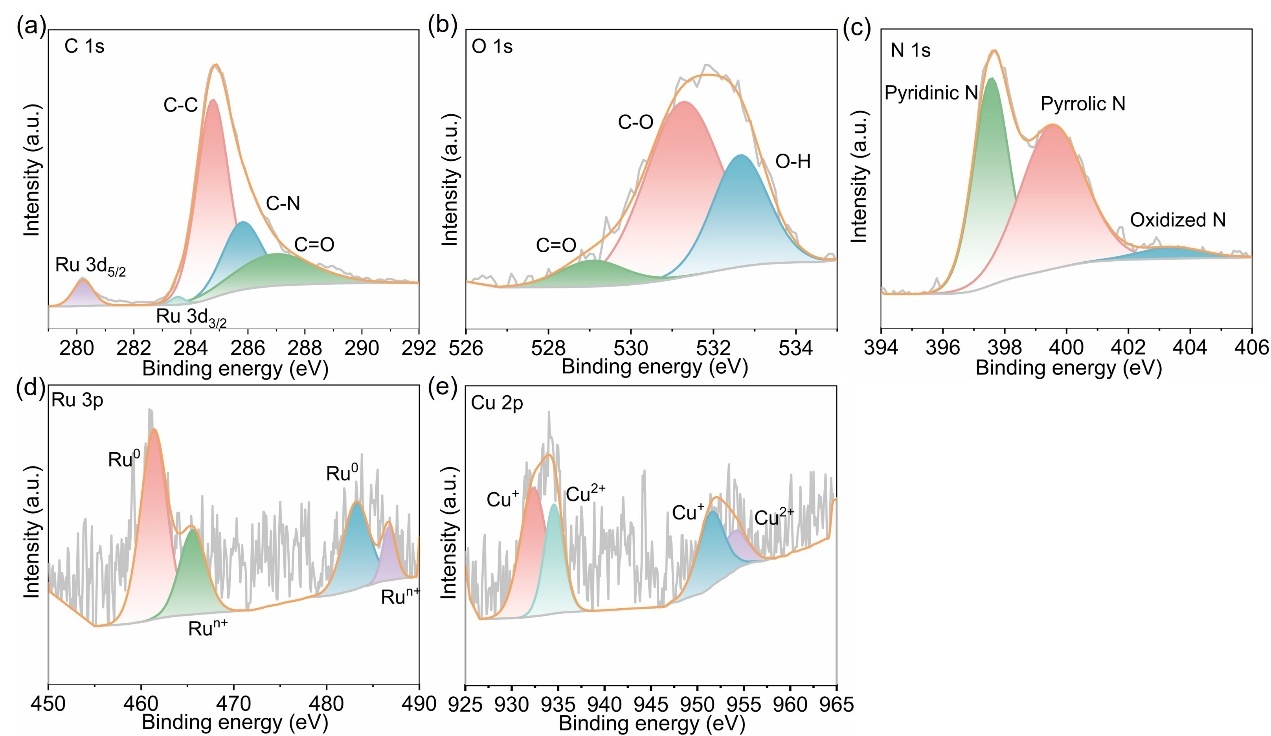


**Fig. S29** High-resolution XPS spectra of RuCu-NC: **a** C 1s, **b** O 1s, **c** N 1s, **d** Ru 3p, and **e** Cu 2p

**
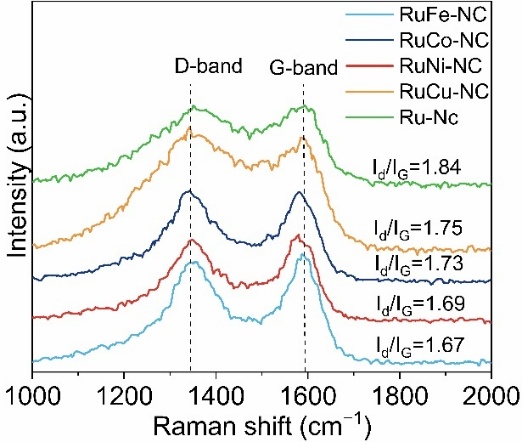
**

**Fig. S30** Raman spectrum of RuFe-NC, RuCo-NC, RuNi-NC and RuCu-NC

**Supplementary Text S4**

As illustrated in Fig. S21, the EDS element mapping images of RuM-NC further disclose that the RuM-NC nanoalloys are well-retained and uniformly dispersed on the porous nitrogen-doped carbon. Furthermore, for all samples, the high-resolution XPS N 1s spectra clearly exhibit peaks corresponding to pyridinic N, pyrrolic N and oxidized N. As depicted in Figs. S25-S29, this observation unequivocally indicates the formation of nitrogen-doped porous carbon substrates. Moreover, the high-resolution XPS O 1s spectra of each sample display three characteristic peaks, which can be attributed to C=O, C−O, and O−H, respectively. It is highly likely that the acid-leaching process, which readily induces the formation of carboxyl and hydroxyl groups on the surface of the carbon substrate, is accountable for this phenomenon. These results strongly demonstrate the formation of RuM-NC nanoalloys that are firmly anchored onto the porous nitrogen-doped carbon substrates.


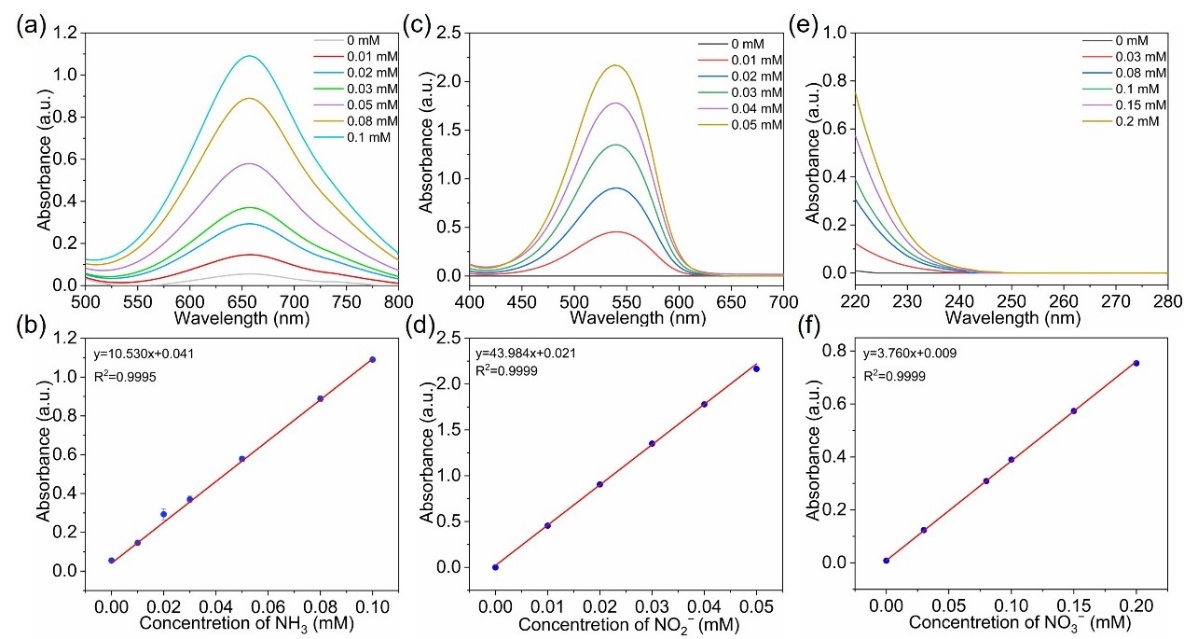


**Fig. S31** Standard calibration curves of **a, b** NH_3_, **c, d** NO_2_^−^, and **e, f** NO_3_^−^ in a 0.5 M K_2_SO_4_ solution. The UV-Vis absorption spectra (top) and corresponding calibration curves (down). The error bars correspond to the standard deviations of three independent measurements


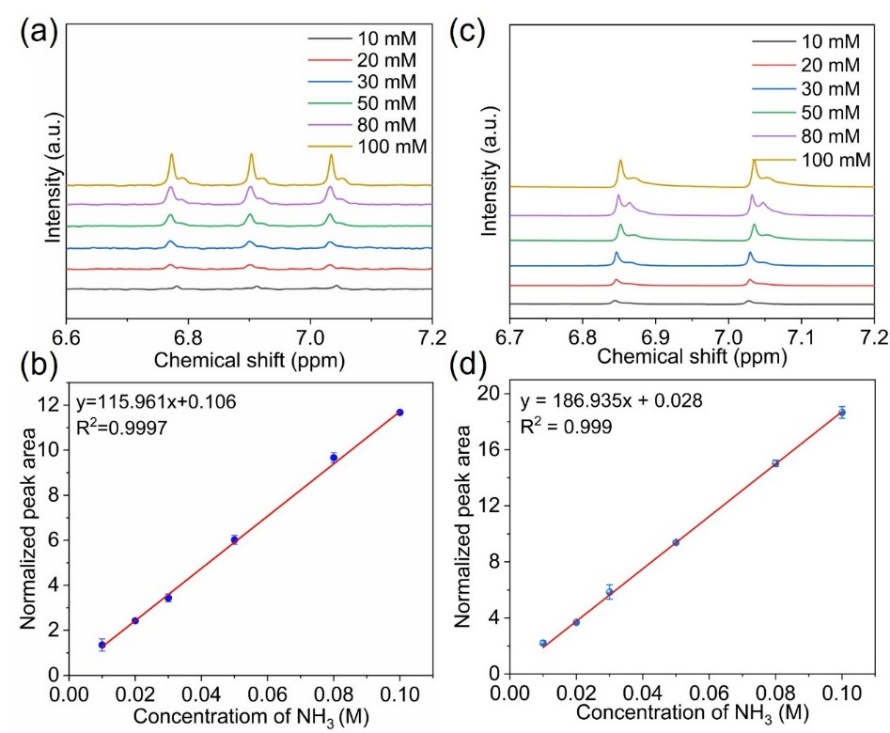


**Fig. S32** Quantification of ammonia using ^1^H NMR spectroscopy for **a, b** ^14^NH_3_ and **c**, **d** ^15^NH_3_ in 0.5 M K_2_SO_4_ aqueous solution. The error bars correspond to standard deviations of three independent measurements


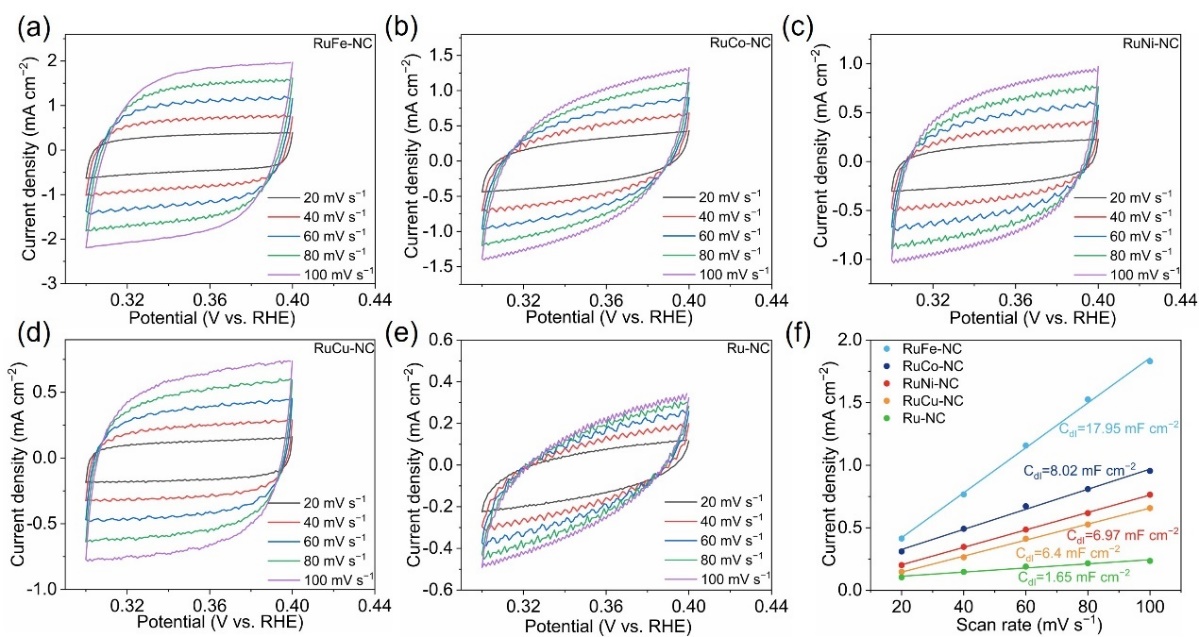


**Fig. S33** CV curves of **a** RuFe-NC, **b** RuCo-NC, **c** RuNi-NC, **d** RuCu-NC, **e** Ru-NC at different scan rates from 20 to 100 mV s^−1^ in the non-faradaic region. **f** The capacitive current differences as a function of the CV scan rates of RuFe-NC, RuCo-NC, RuNi-NC, RuCu-NC, Ru-NC


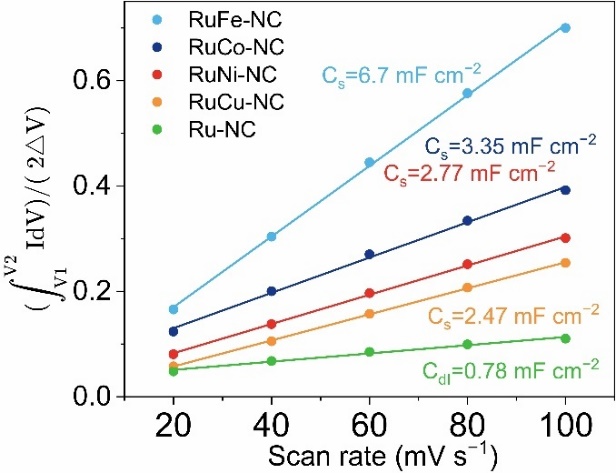


**Fig. S34** The $\int_{V_{1}}^{V_{2}} IdV$as a function of the CV scan rates of RuFe-NC, RuCo-NC, RuNi-NC, RuCu-NC, Ru-NC


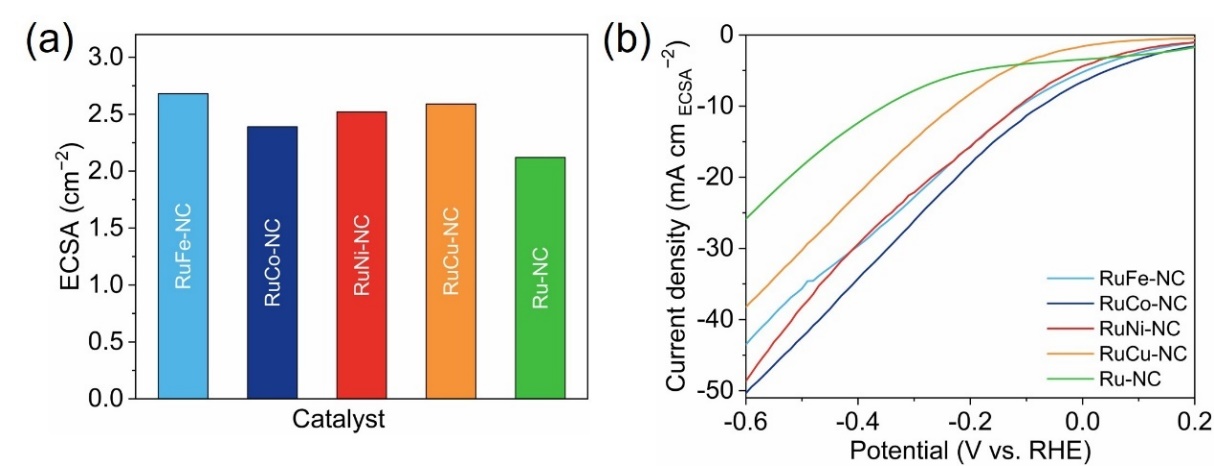


**Fig. S35** **a** ECSA values of RuFe-NC, RuCo-NC, RuNi-NC, RuCu-NC and Ru-NC. **b** LSV curves of RuFe-NC, RuCo-NC, RuNi-NC, RuCu-NC and Ru-NC after normalizing ECSAs in 0.5 M K_2_SO_4_ with 0.1 M KNO_3_


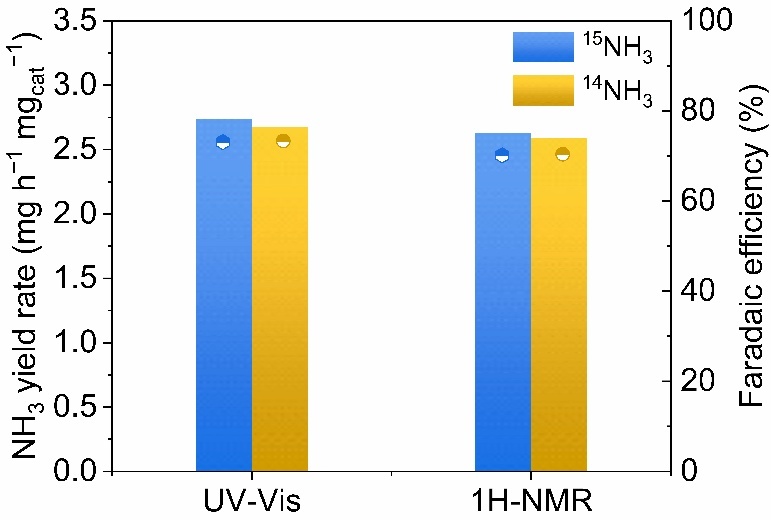


**Fig. S36** NH_3_ yield and FE of RuFe-NC catalyst at −0.5 V vs RHE quantified by ^1^H NMR and UV-Vis


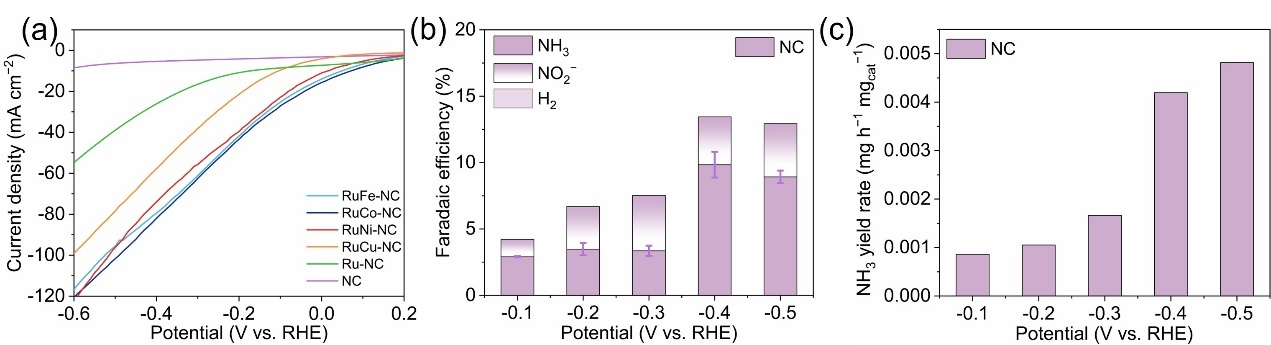


**Fig. S37** **a** LSV curves of RuFe-NC, RuCo-NC, RuNi-NC, RuCu-NC, Ru-NC and NC in 0.5 M K_2_SO_4_ with 0.1 M NO_3_^−^ **b** FE and **c** NH_3_ yield rate of NC in 0.5 M K_2_SO_4_ with 0.1 M NO_3_^−^


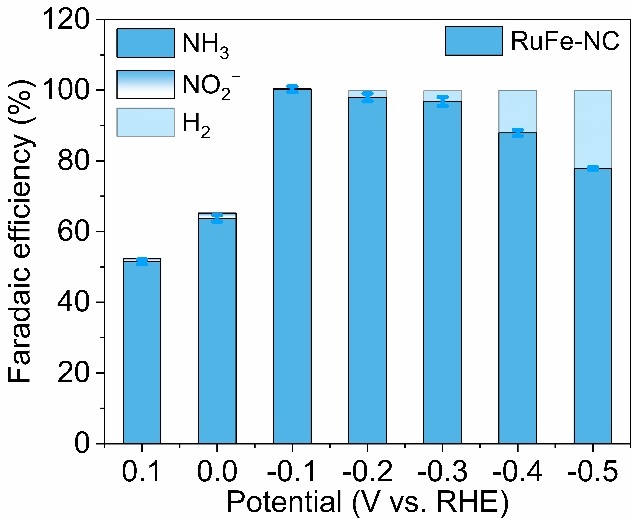


**Fig. S38** FE of RuFe-NC in the potential range of 0.1 to −0.5 V vs. RHE


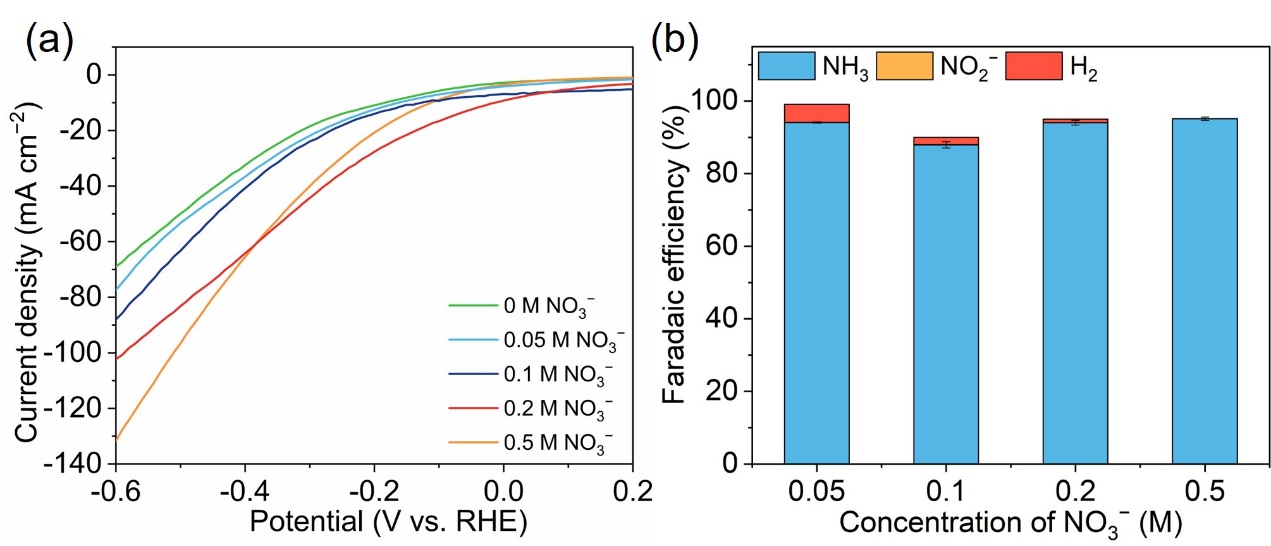


**Fig. S39 a** LSV curve of RuFe-NC under 0.5 M K_2_SO_4_ containing the different concentrations of NO_3_^−^, **b** corresponding FE for NH_3_ production at −0.4 V vs RHE


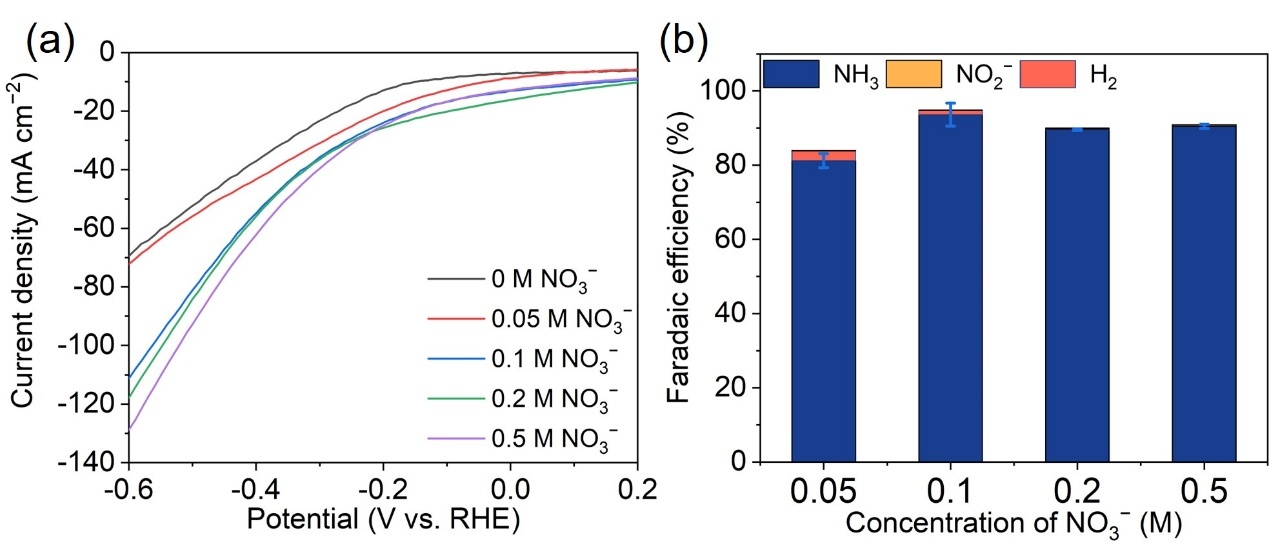


**Fig. S40 a** LSV curve of RuCo-NC under 0.5 M K_2_SO_4_ containing the different concentrations of NO_3_^−^, **b** corresponding FE for NH_3_ production at −0.4 V vs RHE


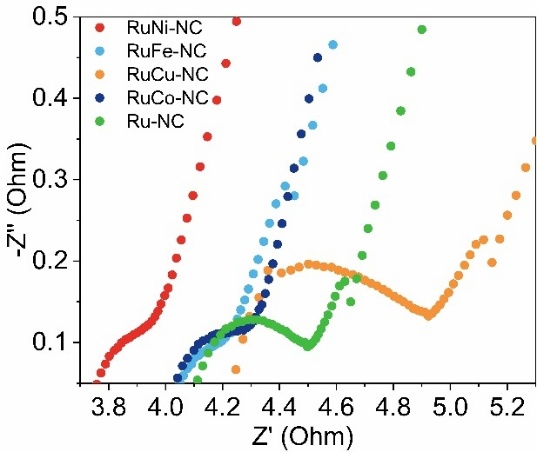


**Fig. S41** The electrochemical impedance spectroscopy of RuFe-NC, RuCo-NC, RuNi-NC, RuCu-NC and Ru-NC


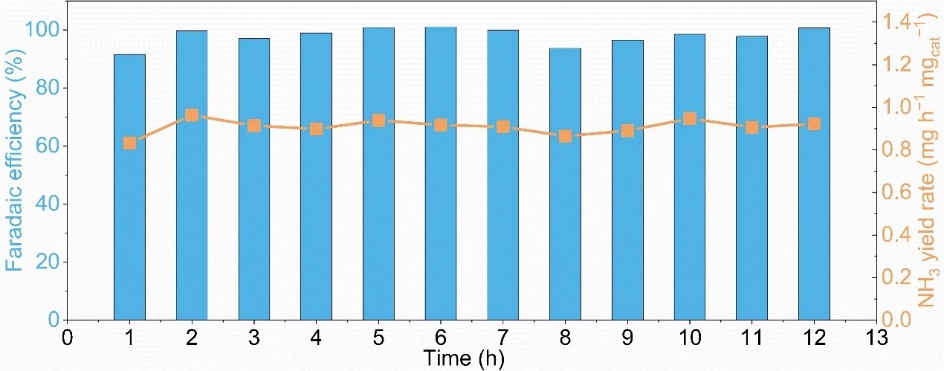


**Fig. S42** The consecutive recycling test at −0.1V vs RHE over RuFe-NC


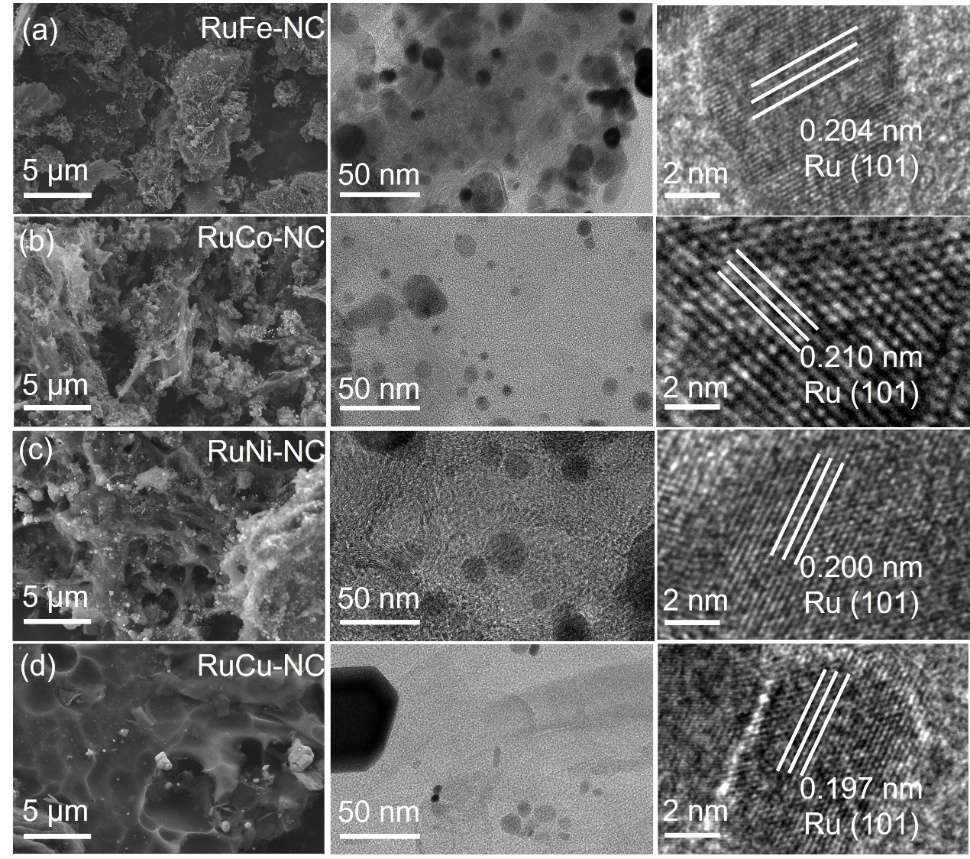


**Fig. S43** The SEM and TEM image of **a** RuFe-NC, **b** RuCo-NC, **c** RuNi-NC and **d** RuCu-NC after the electrolysis


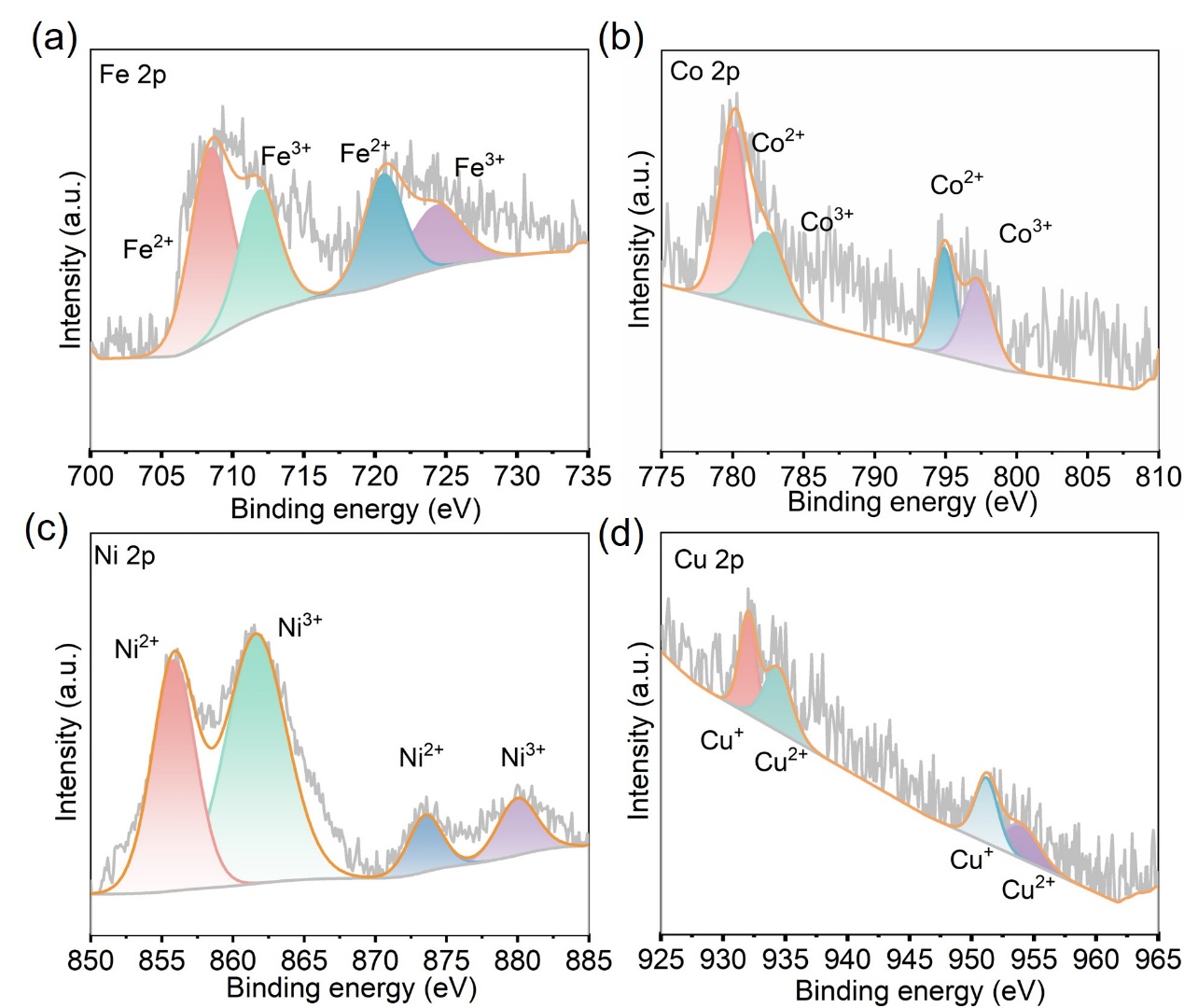


**Fig. S44** High-resolution XPS spectrum after stability testing: **a** Fe 2p, **b** Co 2p, **c** Ni 2p and **d** Cu 2p


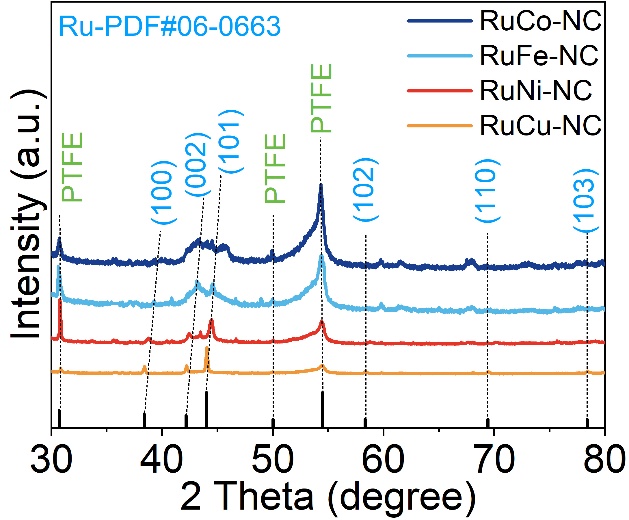


**Fig. S45** XRD pattern of **a** RuFe-NC, **b** RuCo-NC, **c** RuNi-NC, and **d** RuCu-NC samples after stability testing


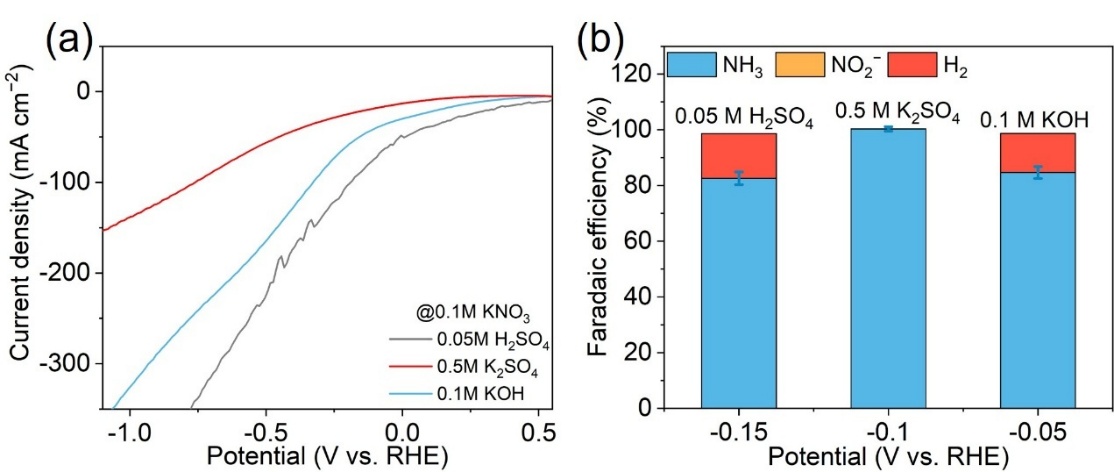


**Fig. S46 a** LSV curve of RuFe-NC under the different electrolytes containing 0.1 M NO_3_^−^, **b** corresponding FE for NH_3_ production under the different applied potentials

To further elucidate the synergistic effect of RuM nanoalloys in electrocatalytic NO_3_RR, we evaluated the performance of RuM-NC across different pH conditions. As illustrated in Fig. S46a, RuM-NC exhibits more favorable onset potentials for NO_3_^−^ electroreduction in both acidic and alkaline media compared to neutral conditions. Notably, while the H_2_ evolution activity increases in extreme pH environments (Fig. S46b), the FE of NH_3_ is still beyond 80% across all tested conditions.


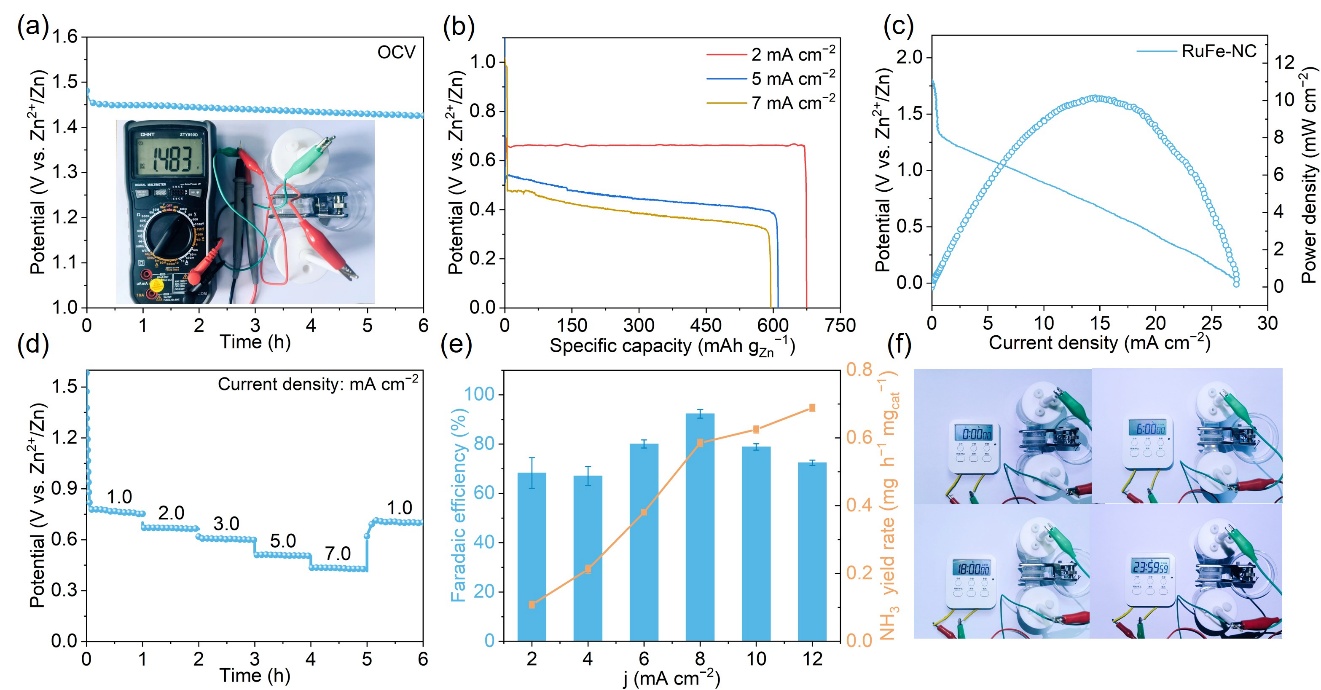


**Fig. S47** Demonstration of Rechargeable Zn-NO_3_^−^ battery. **a** open-circuit potential of the RuFe-NC-based Zn–NO_3_^–^ battery in the rest period, **b** The specific capacities of RuFe-NC-assembled Zn-NO_3_^–^ battery under the current densities of 2, 5 and 7 mA cm^–2^, **c** discharging polarization and power density curves, **d** discharging tests under current densities of Zn–NO_3_^–^ battery, **e** NH_3_ FEs and NH_3_ yield rates in the discharge process, **f** A photograph of an electronic timer powered by the Zn–NO_3_^–^ battery

**Table S1** The element content of RuFe-NC by TEM

| Element | Atomic Fraction (%) | Atomic Error (%) | Mass Fraction (%) | Mass Error (%) |
| --- | --- | --- | --- | --- |
| C | 94.00 | 2.91 | 82.19 | 1.74 |
| N | 0.00 | 0.24 | 0.00 | 0.24 |
| O | 3.70 | 0.75 | 4.31 | 0.87 |
| Fe | 1.03 | 0.14 | 4.19 | 0.56 |
| Ru | 1.27 | 0.19 | 9.31 | 1.41 |

**Table S2** The element content of RuCo-NC by TEM

| Element | Atomic Fraction (%) | Atomic Error (%) | Mass Fraction (%) | Mass Error (%) |
| --- | --- | --- | --- | --- |
| C | 92.31 | 2.74 | 81.04 | 1.57 |
| N | 0.00 | 0.16 | 0.00 | 0.17 |
| O | 5.56 | 1.13 | 6.50 | 1.31 |
| Co | 1.06 | 0.15 | 4.58 | 0.62 |
| Ru | 1.07 | 0.16 | 7.88 | 1.19 |

**Table S3** The element content of RuNi-NC by TEM

| Element | Atomic Fraction (%) | Atomic Error (%) | Mass Fraction (%) | Mass Error (%) |
| --- | --- | --- | --- | --- |
| C | 89.82 | 4.76 | 69.91 | 2.56 |
| N | 0.20 | 0.34 | 0.18 | 0.31 |
| O | 5.82 | 1.20 | 6.04 | 1.22 |
| Ni | 1.23 | 0.18 | 4.69 | 0.65 |
| Ru | 2.93 | 0.46 | 19.18 | 2.94 |

**Table S4** The element content of RuCu-NC by TEM

| Element | Atomic Fraction (%) | Atomic Error (%) | Mass Fraction (%) | Mass Error (%) |
| --- | --- | --- | --- | --- |
| C | 78.56 | 7.49 | 47.39 | 3.11 |
| N | 8.67 | 1.94 | 6.10 | 1.30 |
| O | 4.12 | 0.91 | 3.31 | 0.70 |
| Cu | 0.39 | 0.06 | 1.25 | 0.18 |
| Ru | 8.27 | 1.46 | 41.96 | 6.81 |

**Table S5** The element content of RuM (M=Fe, Co, Ni, Cu)-NC samples by XPS

| Catalyst | Ru (wt%) | C (wt%) | N (wt%) | O (wt%) | Fe (wt%) | Co (wt%) | Ni (wt%) | Cu  (wt%) |
| --- | --- | --- | --- | --- | --- | --- | --- | --- |
| RuFe-NC | 1.2 | 87.07 | 3.41 | 7.39 | 0.94 | / | / | / |
| RuCo-NC | 1.41 | 87.82 | 2.99 | 7.11 | / | 0.67 | / | / |
| RuNi-NC | 1.07 | 86.17 | 6.03 | 5.83 | / | / | 0.91 | / |
| RuCu-NC | 1.12 | 75.72 | 12.65 | 9.95 | / | / | / | 0.56 |
| Ru-NC | 6.38 | 82.31 | 6.46 | 4.86 | / | / | / | / |

**Table S6** The performance of electrocatalytic NO_3_RR for the reported electrocatalysts

| Catalyst | Electrolyte | Onset Potential  (V vs RHE) | Potential  (V vs RHE) | NH_3_ yield  rate | FE (%) | References |
| --- | --- | --- | --- | --- | --- | --- |
| RuFe-NC | 0.5 M K_2_SO_4_ + 0.1 M KNO_3_ | +0.05 | −0.1 | 3.08 mg h^−1^ mg_cat_^−1^ | 100 | This work |
| RuCo-NC | 0.5 M K_2_SO_4_ + 0.1 M KNO_3_ | +0.07 | −0.2 | 3.35 mg h^−1^ mg_cat_^−1^ | 98.62 | This work |
| RuNi-NC | 0.5 M K_2_SO_4_ + 0.1 M KNO_3_ | +0.01 | −0.1 | 2.74 mg h^−1^ mg_cat_^−1^ | 96.85 | This work |
| RuCu-NC | 0.5 M K_2_SO_4_ + 0.1 M KNO_3_ | −0.11 | −0.5 | 2.20 mg h^−1^ mg_cat_^−1^ | 95.60 | This work |
| CoNi@NC | 1 M KOH +  0.1 M KNO_3_ | −0.1 | −0.1 | 168mmol g_cat_^−1^ h^−1^ | 93.00 | [S6] |
| CFP-Cu_1_Ni_1_ | 0.5 M Na_2_SO_4_ + 0.1 M KNO_3_ | +0.1 | −0.22 | 2550 μmol h^−1^ mg_cat_^−1^ | 95.7 | [S7] |
| Pd_74_Ru_26_NCs | 1 M KOH +  32.3 mM KNO_3_ | +0.4 | −0.3 | 16.20 mg h^−1^ cm^−2^ | ∼100 | [S8] |
| CuNi/NC | 0.1 M PBS | −0.3 | −1.0 | / | 94.4 | [S9] |
| RuCo@TDC | 0.1 M KOH + 0.1 M KNO_3_ | +0.2 | −0.2 | 0.665 mmol h^−1^ cm^−2^ | 95.7 | [S10] |
| Ru_5_Mo_5_-NC | 0.5 M K_2_SO_4_ + 0.1 M KNO_3_ | −0.05 | −0.4 | 1.3 mg h^−1^ mg_cat_^−1^ | 98.3 | [S11] |
| Bi_1_Pd | 0.1 M KOH +0.1 M KNO_3_ | −0.2 | −0.6 | 33.8 mg h^–1^ cm^–2^ | 99.6 | [S12] |
| Co@CC/CP | 0.1 M NaOH + 0.1 M KNO_3_ | +0.1 | −0.8 | 0.60 mmol h^−1^ cm^−2^ | 93.4 | [S13] |
| FeIr | 0.5 M K_2_SO_4_ + 1000 ppm KNO_3_ | −0.1 | −0.6 | 11.67 mg h^–1^ cm^–2^ | 97.3 | [S14] |
| RhCu NCs | 0.5 M Na_2_SO_4_ + 0.1 M KNO_3_ | +0.27 | −0.1 | 8.1 mg h^−1^ mg_cat_^−1^ | 97.5 | [S15] |
| Au_3_Cu/CC | 0.5 M Na_2_SO_4_ +0.05 M KNO_3_ | −0.05 | −0.5 | 1719.3 μg h^−1^ cm^−2^ | 95.1 | [S16] |

**Supplementary References**

[S1] C. Liu;M. Hirohara;T. Maekawa;R. Chang;T. Hayashi;C.-Y. Chiang, Selective Electro-Oxidation of Glycerol to Dihydroxyacetone by a Non-Precious Electrocatalyst–CuO. Appl. Catal. B-Environ. **265**(118543) (2020). <https://doi.org/10.1016/j.apcatb.2019.118543>

[S2] C. Zhang;R. Lu;C. Liu;J. Lu;Y. Zou;L. Yuan;J. Wang;G. Wang;Y. Zhao;C. Yu, Trimetallic Sulfide Hollow Superstructures with Engineered D-Band Center for Oxygen Reduction to Hydrogen Peroxide in Alkaline Solution. Adv. Sci. **9**(12), 2104768 (2022). <https://doi.org/10.1002/advs.202104768>

[S3] K.W. Jacobsen;J.K. Norskov;M.J. Puska, Interatomic Interactions in the Effective-Medium Theory. Phys. Rev. B. **35**(14), 7423-7442 (1987). <https://doi.org/10.1103/PhysRevB.35.7423>

[S4] J.R. Kitchin;J.K. Nørskov;M.A. Barteau;J.G. Chen, Modification of the Surface Electronic and Chemical Properties of Pt(111) by Subsurface 3d Transition Metals. J. Chem. Phys. **120**(21), 10240-10246 (2004). <https://doi.org/10.1063/1.1737365>

[S5] W. Gao;K. Xie;J. Xie;X. Wang;H. Zhang;S. Chen;H. Wang;Z. Li;C. Li, Alloying of Cu with Ru Enabling the Relay Catalysis for Reduction of Nitrate to Ammonia. Adv. Mater. **35**(19), 2202952 (2023). <https://doi.org/10.1002/adma.202202952>

[S6] F. Lei;Y. Zhang;M. Xu;K. Li;M. Zhang;R. Huai;J. Xie;P. Hao;G. Cui;B. Tang, Energy-Efficient Ammonia Synthesis from Nitrate Via CoNi Alloys Incorporated in Carbon Frameworks. ACS Sustain. Chem. Eng. **11**(24), 9057-9064 (2023). <https://doi.org/10.1021/acssuschemeng.3c01688>

[S7] Z. Zhang;Y. Liu;X. Su;Z. Zhao;Z. Mo;C. Wang;Y. Zhao;Y. Chen;S. Gao, Electro-Triggered Joule Heating Method to Synthesize Single-Phase CuNi Nano-Alloy Catalyst for Efficient Electrocatalytic Nitrate Reduction toward Ammonia. Nano Res. **16**(5), 6632-6641 (2023). <https://doi.org/10.1007/s12274-023-5402-y>

[S8] Y. Hu;J. Liu;W. Luo;J. Dong;C. Lee;N. Zhang;M. Chen;Y. Xu;D. Wu;M. Zhang;Q. Zhu;E. Hu;D. Geng;L. Zhong;Q. Yan, Alloying Pd with Ru Enables Electroreduction of Nitrate to Ammonia with ∼100% Faradaic Efficiency over a Wide Potential Window. Chem. Sci. **15**(21), 8204-8215 (2024). <https://doi.org/10.1039/D4SC00558A>

[S9] Y. Liu;B. Deng;K. Li;H. Wang;Y. Sun;F. Dong, Metal-Organic Framework Derived Carbon-Supported Bimetallic Copper-Nickel Alloy Electrocatalysts for Highly Selective Nitrate Reduction to Ammonia. J. Colloid Int. Sci. **614**, 405-414 (2022). <https://doi.org/10.1016/j.jcis.2022.01.127>

[S10] S. Wu;J. Yan;D. Zhao;Z. Cai;J. Yu;R. Li;Q. Li;G. Fan, Three-Dimensional RuCo Alloy Nanosheets Arrays Integrated Pinewood-Derived Porous Carbon for High-Efficiency Electrocatalytic Nitrate Reduction to Ammonia. J. Colloid Int. Sci. **668**, 264-271 (2024). <https://doi.org/10.1016/j.jcis.2024.04.145>

[S11] X. Ge;R. Pan;H. Xie;S. Hu;J. Yuan, Regulating Ruxmoy Nanoalloys Anchored on Porous Nitrogen-Doped Carbon Via Domain-Confined Etching Strategy for Neutral Efficient Ammonia Electrosynthesis. Nano Lett. **24**(39), 12218-12225 (2024). <https://doi.org/10.1021/acs.nanolett.4c03319>

[S12] K. Chen;Z. Ma;X. Li;J. Kang;D. Ma;K. Chu, Single-Atom Bi Alloyed Pd Metallene for Nitrate Electroreduction to Ammonia. Adv. Funct. Mater. **33**(12), 2209890 (2023). <https://doi.org/10.1002/adfm.202209890>

[S13] T. Xie;X. Li;J. Li;J. Chen;S. Sun;Y. Luo;Q. Liu;D. Zhao;C. Xu;L. Xie;X. Sun, Co Nanoparticles Decorated Corncob-Derived Biomass Carbon as an Efficient Electrocatalyst for Nitrate Reduction to Ammonia. Inorg. Chem. **61**(35), 14195-14200 (2022). <https://doi.org/10.1021/acs.inorgchem.2c02499>

[S14] J. Xiong;L. Jiang;B. Zhu;S. Huang;S. Wu;K. You;X. Li;L. Feng, Feir Alloy Optimizes the Trade-Off between Nitrate Reduction and Active Hydrogen Generation for Efficient Electro-Synthesis of Ammonia in Neutral Media. Adv. Funct. Mater. 2423705 (2025). <https://doi.org/10.1002/adfm.202423705>

[S15] L. Zhu;H. Yao;L. Sun;L. Ai;H. Zhai;C. Yi, Alloyed Rhodium-Copper Nanocavities with Optimized Chemisorption of Hydrogen Radicals for Efficient Nitrate-to-Ammonia Electrocatalysis. Small. **21**(19), 2502787 (2025). <https://doi.org/10.1002/smll.202502787>

[S16] S. Sun;D. Zhang;D. Li;C. Xu;H. Mou;L. Feng;S. Lv;C. Song;J. Song;D. Wang, Boosting Nitrate Electroreduction to Ammonia on Alloying of Cu with Au by Accelerated Proton Relay. Agg. **6**(5), e70016 (2025). <https://doi.org/10.1002/agt2.70016>
